# Degenerate Pax2 and Senseless binding motifs improve detection of low-affinity sites required for enhancer specificity

**DOI:** 10.1371/journal.pgen.1007289

**Published:** 2018-04-04

**Authors:** Arya Zandvakili, Ian Campbell, Lisa M. Gutzwiller, Matthew T. Weirauch, Brian Gebelein

**Affiliations:** 1 Graduate Program in Molecular and Developmental Biology, Cincinnati Children's Hospital Research Foundation, Cincinnati, OH, United States of America; 2 Medical-Scientist Training Program, University of Cincinnati College of Medicine, Cincinnati, OH, United States of America; 3 Division of Developmental Biology, Cincinnati Children’s Hospital, MLC, Cincinnati, OH, United States of America; 4 Center for Autoimmune Genomics and Etiology & Division of Biomedical Informatics, Cincinnati Children’s Hospital, MLC, Cincinnati, OH, United States of America; 5 Department of Pediatrics, University of Cincinnati College of Medicine, Cincinnati, OH, United States of America; University of Michigan Medical School, UNITED STATES

## Abstract

Cells use thousands of regulatory sequences to recruit transcription factors (TFs) and produce specific transcriptional outcomes. Since TFs bind degenerate DNA sequences, discriminating functional TF binding sites (TFBSs) from background sequences represents a significant challenge. Here, we show that a *Drosophila* regulatory element that activates Epidermal Growth Factor signaling requires overlapping, low-affinity TFBSs for competing TFs (Pax2 and Senseless) to ensure cell- and segment-specific activity. Testing available TF binding models for Pax2 and Senseless, however, revealed variable accuracy in predicting such low-affinity TFBSs. To better define parameters that increase accuracy, we developed a method that systematically selects subsets of TFBSs based on predicted affinity to generate hundreds of position-weight matrices (PWMs). Counterintuitively, we found that degenerate PWMs produced from datasets depleted of high-affinity sequences were more accurate in identifying both low- and high-affinity TFBSs for the Pax2 and Senseless TFs. Taken together, these findings reveal how TFBS arrangement can be constrained by competition rather than cooperativity and that degenerate models of TF binding preferences can improve identification of biologically relevant low affinity TFBSs.

## Author summary

While all cells in an organism share a common genome, each cell type must express the appropriate combination of genes needed for its specific function. Cells activate and repress different parts of the genome using transcription factor proteins that bind regulatory regions known as enhancers. We currently have an incomplete view of how enhancers recruit transcription factors to yield accurate gene activation and repression. This problem is complicated by the fact that most animals contain over a thousand different transcription factors, and each can generally bind multiple DNA sequences. Thus, it is difficult to predict which transcription factors interact with which enhancers. To gain insights into this process, we focused on determining how an enhancer that activates a gene needed to make liver-like cells is regulated in a precise manner in the fruit-fly embryo. We demonstrate that the specific activity of this enhancer depends on weak and overlapping transcription factor binding sites. Furthermore, we demonstrate that computational models that include weak transcription factor interactions yield better predictive accuracy. These results shed light on how DNA sequences determine enhancer activity and the types of strategies that are most useful for predicting transcription factor binding sites in the genome.

## Introduction

The control of gene expression is fundamental for defining a cell’s identity and ability to respond to environmental cues. At the transcriptional level, *cis*-regulatory modules (CRMs) act as platforms for transcription factors (TFs) that affect RNA polymerase activity [1, 2]. Hence, the number, organization, and affinity of TF binding sites (TFBSs) within a CRM convert information about cellular context conveyed by TFs into transcriptional activity [1, 3]. A typical strategy for predicting TFBSs is to use a model of TF binding specificity, such as a position-weight matrix, to score sequences and those with higher scores are predicted to have a greater likelihood of being functional TFBSs. However, this approach is called into question by the growing literature that reveals suboptimal TFBSs are often necessary for accurate biological function [4–11].

Evidence supporting biological relevance of suboptimal TFBSs can be summarized using four concepts [4, 12]. First, suboptimal TFBSs are more likely to differentiate between TFs with similar binding preferences. For instance, suboptimal Hox binding sites were empirically identified in the *Drosophila shavenbaby (svb)* enhancer and the non-consensus nature of these sites was critical to ensure *svb* is activated by abdominal, but not thoracic Hox factors [5]. Second, suboptimal TFBSs can be more sensitive to context (e.g. TF concentration). In a classic example, *Caenorhabditis elegans* genes associated with high-affinity PHA-4 TFBSs are expressed earlier in development when PHA-4 levels are low, whereas genes with low-affinity PHA-4 sites are induced by higher PHA-4 levels later in development [6]. Third, TFBS affinity can alter the ability of a TF to either activate or repress transcription. For example, *Drosophila* Hedgehog-responsive CRMs with a cluster of low-affinity Ci TFBSs activate transcription, whereas increasing the affinity of Ci TFBSs resulted in repression [7]. Fourth, CRM specificity may depend on suboptimal interactions between TFs. For instance, reporter assays interrogating the Otx-a enhancer in *Ciona* revealed suboptimal spacing between TFBSs promote enhancer specificity [8, 9]. These studies collectively demonstrate that low-affinity interactions between TFs and CRMs play an important role in accurate transcriptional regulation.

Since TFs have degenerate binding preferences and suboptimal sites are often biologically relevant, predicting functional TFBSs from background sequence is challenging. TFBS-prediction algorithms are typically binary classifiers: sequences are scored using a model of TF binding specificity (e.g. a PWM) and those that meet a threshold are classified as TFBSs. Moreover, the field has largely used arbitrary thresholds as default settings for TFBS-prediction algorithms, such as the 0.8 relative log-likelihood threshold—e.g. a recommended default on the JASPAR website [13]. How well these standard thresholds identify suboptimal TFBSs remains unclear, and the cost of lowering thresholds to identify suboptimal TFBSs (i.e. increased false-discovery rate) is largely unknown.

**Fig 1:**
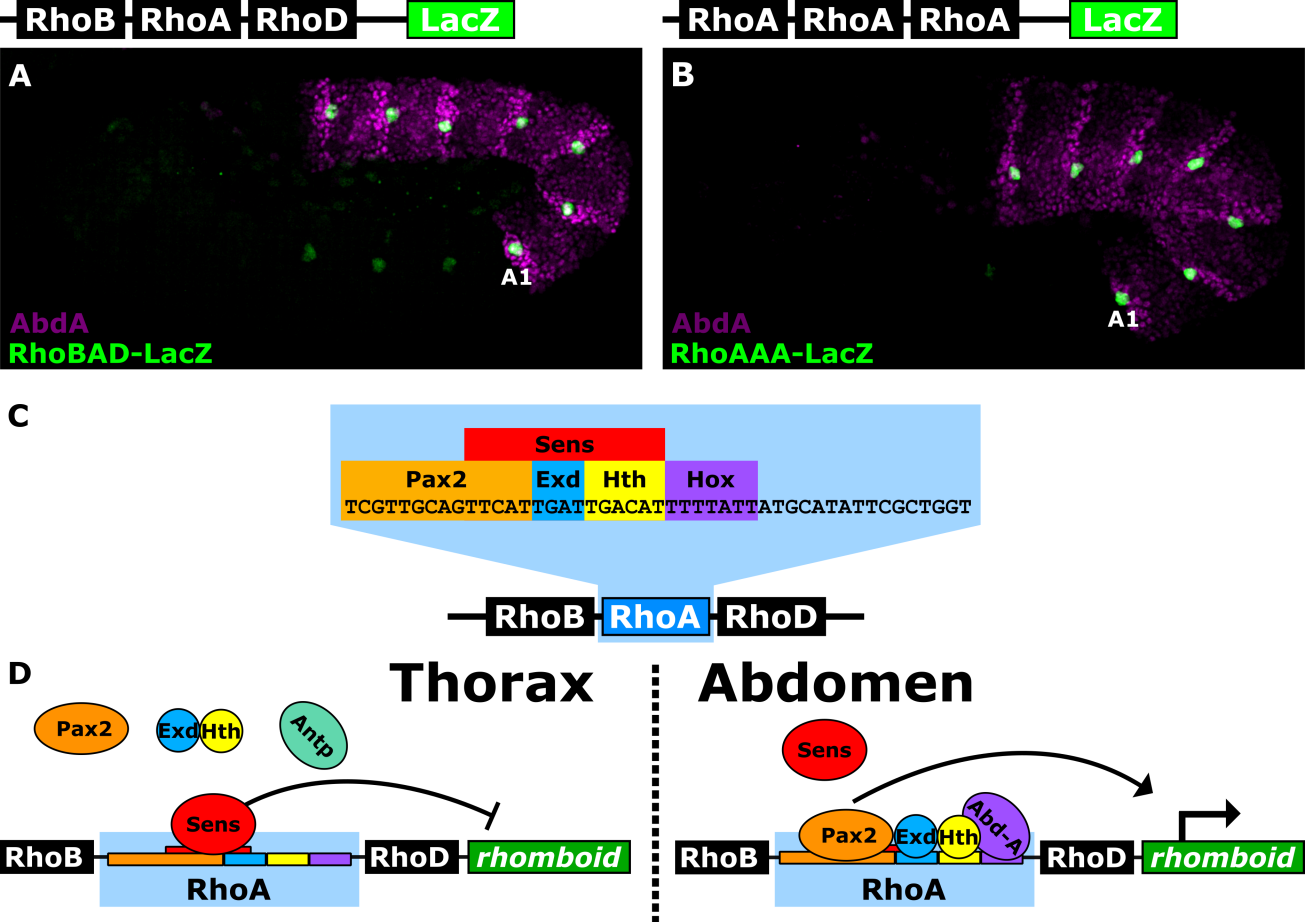
The *RhoA* enhancer activates gene expression in *Drosophila* abdominal C1-SOPs. **(A, B)** Lateral view of *Drosophila RhoBAD-LacZ* (A) or *RhoAAA-LacZ* (B) embryos (stage 11) immunostained for β-gal (green) and AbdA (purple). Both reporters are active in a specific cell type (C1-SOP) with higher levels observed in abdominal segments (stained by AbdA, first abdominal segment marked by “A1”) than thoracic segments. **(C)** The *RhoA* sequence has binding sites for Pax2, Sens, Exd, Hth, and AbdA that are critical for proper *RhoBAD-LacZ* and *RhoAAA-LacZ* activity in *Drosophila* embryos [17, 22]. **(D)** Schematic model of competition between activator (Pax2/Exd/Hth/AbdA) and repressor (Sens) TFs. Sens binds and represses *RhoA* activity in the thorax; whereas AbdA and the activators outcompete Sens to promote gene activation in C1-SOP cells of the abdomen.

In this study, we used a well-characterized *Drosophila* CRM, *Rhomboid-BAD* (*RhoBAD*), to assess the role of suboptimal TFBSs for accurate gene regulation and tested the ability of algorithms to predict such sites. The *rhomboid (rho)* gene encodes a serine protease that triggers secretion of an Epidermal Growth Factor (EGF) ligand [14]. *RhoBAD* activates *rho* within specific abdominal sensory organ precursors (C1-SOPs), and thereby induces neighboring cells to form hepatocyte-like cells (oenocytes) essential for animal growth [15–20]. *RhoBAD* specificity is largely defined by a conserved 47 base-pair sequence (*RhoA*) that recruits activating and repressing TFs. Indeed, three copies of *RhoA* are sufficient to recapitulate the abdominal and C1-SOP specific activity of *RhoBAD* (**Fig 1A and 1B**) [21, 22]. In the abdomen, an Abdominal-A (Abd-A) Hox factor and the Extradenticle (Exd) and Homothorax (Hth) homeodomain proteins form a complex with the Pax2 TF to activate gene expression [22]. Thoracic segments, however, lack Abd-A expression and thereby allow the Senseless (Sens) TF to bind and repress *RhoBAD* [17]. Importantly, Pax2 and Sens expression are largely restricted to peripheral nervous system (PNS) cells in all segments. Thus, *RhoA* integrates both segment (Abd-A) and tissue-specific (Sens and Pax2) inputs to ensure accurate expression in abdominal C1-SOPs (**Fig 1C and 1D**).

Here, we show that *RhoBAD* requires overlapping low-affinity TFBSs for Pax2 and Sens to mediate accurate cell- and segment-specific output. Using transgenic reporters and DNA binding assays, we found that increasing Pax2 affinity results in gene activation in additional abdominal PNS cells, whereas increasing Sens affinity results in inappropriate repression. In addition, altering the TFBSs to allow simultaneous binding of activators and repressors impairs *RhoBAD* activity. Testing available TF binding preference models, however, revealed high degrees of variability in predicting these low affinity TFBSs. To define the source of this discrepancy, we developed a method that generates hundreds of PWMs by selectively sampling TFBSs based on predicted affinity. Surprisingly, we found that PWMs created from datasets depleted of high affinity sites were more accurate at predicting both low- and high-affinity Pax2 and Sens TFBSs from bacterial 1-hybrid (B1H), protein binding microarray (PBM), and mammalian ChIP-seq data than PWMs derived from high affinity sites. Altogether, these findings provide new insights into the functional roles of low affinity DNA binding sites and our ability to use computational approaches to identify TFBSs in complex datasets.

## Results

### *RhoA* contains low-affinity Pax2 and Sens TFBSs

**Fig 2:**
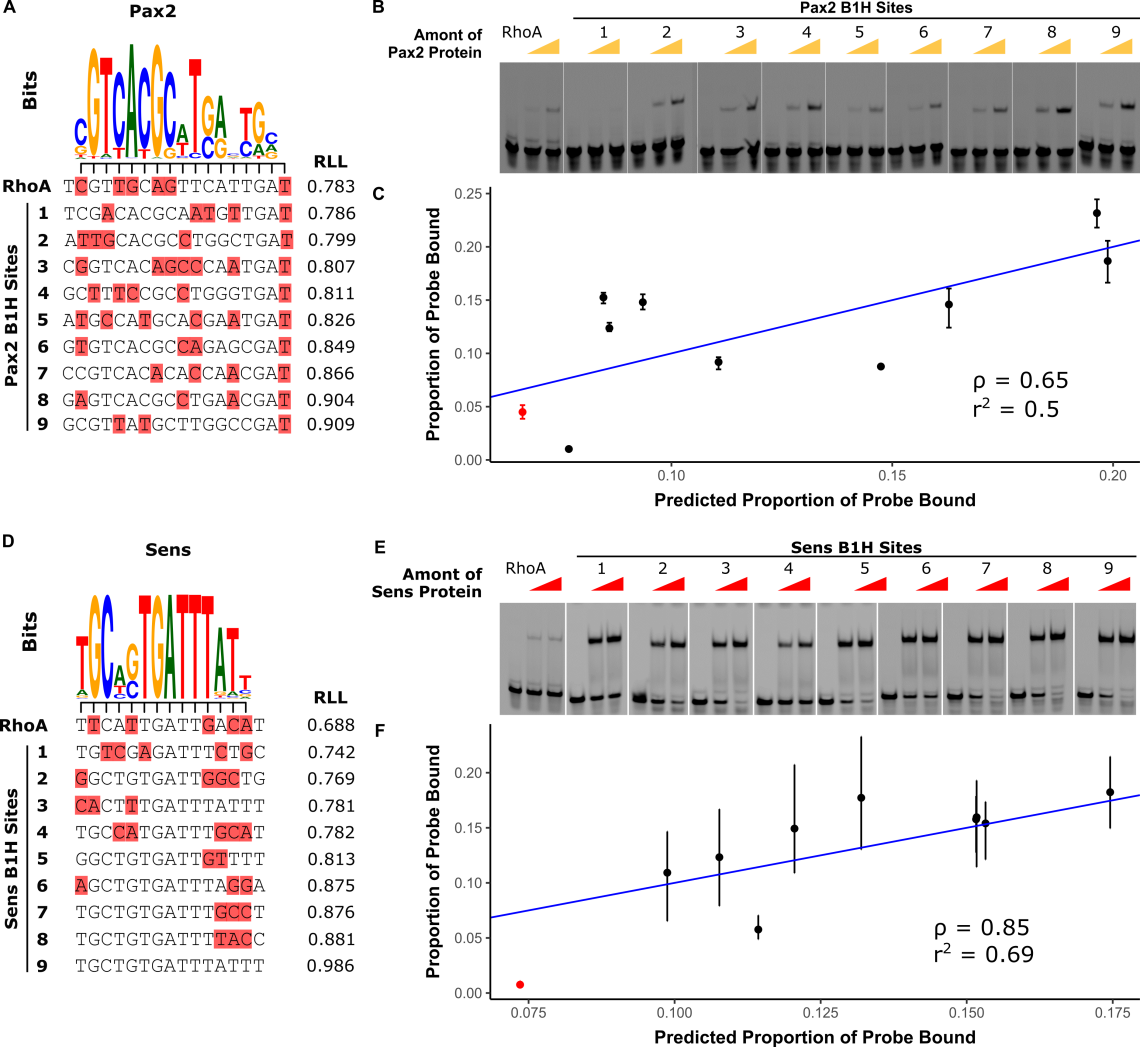
*RhoA* contains low affinity Pax2 and Sens binding sites. **(A, D)** Alignment of Pax2 (A) and Sens (D) logos derived from SELEX-seq [23] to *RhoA* and selected B1H sites [24]. Mismatches to the logos are highlighted in red. **(B, E)** Pax2 (B) and Sens (E) binding to *RhoA* and selected B1H hits using EMSAs. Each probe was incubated with 0, 106, or 212 ng of Sens or 0, 48, or 96 ng of Pax2. Full gels are shown in **S2 Fig**. **(C, F)** Correlation between proportion of probe bound in EMSAs versus proportion predicted by PWM energy models. The Spearman-rank correlation (ρ) and coefficient-of-determination (r^2^) are indicated on the plots. Linear regression of this relationship is shown in blue.

While empirical studies showed that five different transcription factors directly regulate *RhoBAD*, PWMs derived from published SELEX-seq assays [23] fail to predict the Sens and Pax2 TFBSs using the 0.8 relative-to-range log-likelihood (RLL) threshold (default setting on JASPAR [13]) (**Fig 2A and 2D** and **S1 Fig**). This finding suggests that the *RhoA* Sens and Pax2 TFBSs are low affinity and that the PWMs developed using these *in vitro* assays maybe too restrictive to accurately predict such functional low affinity TFBSs. To ascertain how affinity correlates with PWM RLL scores, we used electromobility shift assays (EMSAs) with purified Sens and Pax2 proteins to compare *RhoA* binding to nine randomly selected Pax2 and Sens sites from a published bacterial-1-hyrbid (B1H) study [24] (**Fig 2B and 2E** and **S2 Fig**). The selected B1H sites have a large range of RLL scores (**Fig 2A and 2D)** and were placed in the context of *RhoA* to maintain consistent flanking nucleotides. EMSAs revealed that the PWMs performed well in ranking TFBS affinity with Spearman’s rank correlations (ρ) of 0.65 and 0.85 for Pax2 and Sens, respectively, between predicted and observed binding (see **Methods** for details [25–27]) (**Fig 2C and 2F**). Moreover, these results revealed that the *RhoA* Pax2 and Sens sites (red in **Fig 2C and 2F**) were relatively low in affinity compared to the B1H sites (**Fig 2B, 2C, 2E and 2F**).

### Increasing Pax2 affinity results in ectopic activity within the abdominal PNS

**Fig 3:**
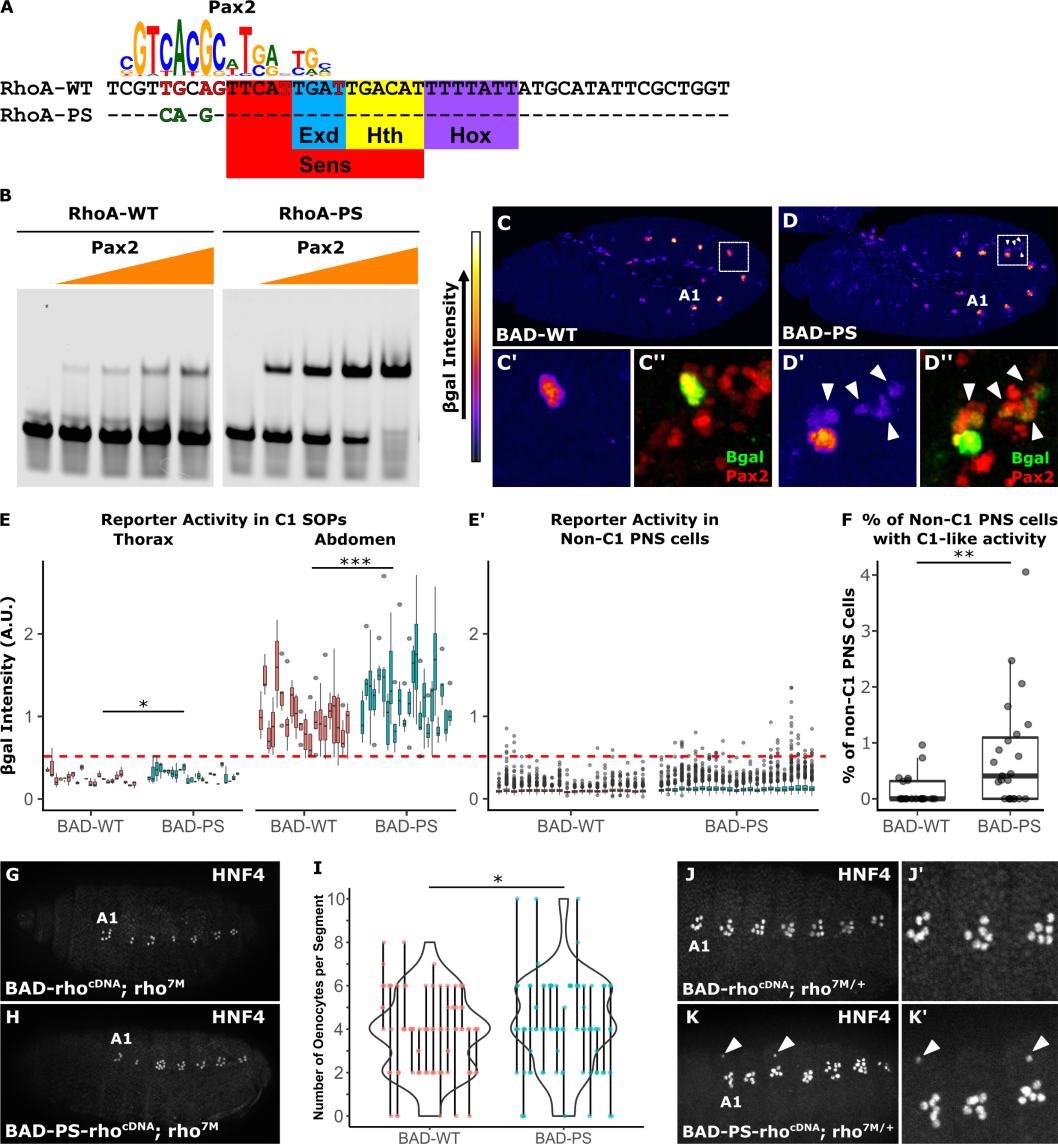
A high affinity Pax2 binding site results in ectopic *RhoA* activity in additional PNS cells. **(A)** The SELEX-seq [23] Pax2 logo aligned with *RhoA-WT* and *RhoA-PS*. Mis-matches are in red font, and nucleotides that improve the match are in green font. The Sens, Exd, Hth, and Hox TFBSs are highlighted. **(B)** EMSAs using purified Pax2 protein (0, 10.25, 20.5, 41, and 82 ng) on *RhoA-WT* and *RhoA-PS* probes reveal Pax2 has a higher affinity for *RhoA-PS* (Full gels are shown in **S3 Fig**). **(C, D)** Lateral view of *RhoBAD-LacZ* (C) and *RhoBAD-PS-lacZ* (D) embryos (stage 11) immunostained for β-gal. β-gal intensity is represented by heat-map at left. “A1” indicates the first abdominal segment. **(C’, D’)** Close-up of an abdominal C1-SOP with arrowheads highlighting non-C1-SOPs that activate *RhoBAD-PS-lacZ*. **(C”, D”**) Same close-up showing β-gal (green) and Pax2 (red). **(E)** Boxplot of β-gal immunostain intensities in thoracic and abdominal C1-SOPs. One-tailed Welch’s t-test was used to compare mean β-gal intensity per embryo (* p < 0.05, *** p < 0.001), n = 20 (WT) and 23 (PS). Each box represents measurements from a single embryo. **(E’)** Boxplot of β-gal immunostain intensities in non-C1 PNS (Sens+) cells. Dotted line represents 5^th^ percentile of β-gal intensity in C1-SOPs–a threshold to define “C1-like reporter activity”. **(F)** Proportion of non-C1 PNS cells per embryo with C1-like β-gal intensities in *RhoBAD-lacZ* and *RhoBAD-PS-lacZ* embryos (** p < 0.01, One-tailed Wilcoxon Rank Sum Test), n = 20 (WT) and 23 (PS). **(G-H)** Lateral view of *RhoBAD-rho*^*cDNA*^*; rho*^*7M*^ (G) and *RhoBAD-PS-rho*^*cDNA*^*; rho*^*7M*^ (H) embryos (stage 15) immunostained for an oenocyte marker (HNF4). Note, in the absence of *rho*, embryos do not develop HNF4+ oenocytes [15, 17]. **(I)** Violin plots of the number of oenocytes (HNF4+) per embryonic segment for all *RhoBAD-rho*^*cDNA*^ or *RhoBAD-PS-rho*^*cDNA*^ embryos. Lines represent range of oenocytes per segment for each embryo, while dots represent individual segments (* p < 0.05, One-tailed Wilcoxon Rank Sum Test), n = 95 (WT) and 98 (PS). **(J, K)** Lateral views of *RhoBAD-rho*^*cDNA*^*; rho*^*7M/+*^ (J) and *RhoBAD-PS-rho*^*cDNA*^*; rho*^*7M/+*^ (K) embryos immunostained for HNF4. Arrowheads indicate ectopic HNF4+ cells. (**J’, K’**) Close-up of A1-A3 abdominal segments of panels J and K.

To determine if *RhoBAD* activity depends on a low-affinity Pax2 TFBS, we altered the Pax2 site to better match the consensus motif (*RhoA-PS*, **Fig 3A**). EMSAs using Pax2 confirmed a greater affinity for *RhoA-PS* than wildtype *RhoA*, without affecting Sens or Exd/Hth/AbdA binding (**Fig 3B,** and **S3 Fig**). Next, we integrated *RhoBAD-lacZ* and *RhoBAD-PS-lacZ* into identical loci and performed quantitative analysis on age-matched *Drosophila* embryos. Like *RhoBAD-lacZ*, *RhoBAD-PS-lacZ* drives high β-gal levels in abdominal C1-SOPs and weak levels in thoracic C1-SOPs, but with a small, statistically significant increase in all segments (**Fig 3C–3E**). In addition, *RhoBAD-PS-lacZ* embryos inappropriately increased β-gal expression in non-C1 PNS cells (**Fig 3D**, arrowheads). To determine if the ectopic activation of *RhoBAD-PS* reaches an "abdominal C1-SOP-like" level of activity, we defined a threshold equal to the 5^th^ percentile of wild type *RhoBAD-lacZ* abdominal C1-SOP intensity and above the 100^th^ percentile of thoracic C1-SOP intensity (red line in **Fig 3E–3E'**). Using this threshold, we found that β-gal is ectopically expressed in over 5 times more PNS cells in *RhoBAD-PS-lacZ* than *RhoBAD-lacZ* embryos (**Fig 3F**). As a control, no difference in intensity of Sens staining was observed in these embryos (**S4 Fig**). Thus, strengthening Pax2 binding results in increased *RhoBAD* activity within C1-SOPs as well as in additional abdominal PNS cells.

To assess if the increased activity of *RhoBAD-PS* can have functional consequences, we developed an oenocyte rescue assay. In the absence of *rho*, no EGF signal is sent from abdominal C1-SOPs, and thus adjacent ectodermal cells fail to differentiate into oenocytes [15, 17]. However, *rho* mutant embryos (*rho*^*7M*^) carrying a wild type *RhoBAD-rho*^*cDNA*^ transgene can substantially rescue oenocyte (HNF4+) formation (**Fig 3G**) [18, 28]. Consistent with *RhoBAD-PS* having increased reporter activity, *RhoBAD-PS-rho*^*cDNA*^ induced a significant increase in oenocyte numbers (**Fig 3H and 3I**). Moreover, analysis of *rho* heterozygous embryos carrying *RhoBAD-PS-rho*^*cDNA*^ revealed that 30% (3/10) of the embryos had at least one segment with ectopic oenocytes whereas none were observed in embryos with wild type *RhoBAD-rho*^*cDNA*^ (arrowheads in **Fig 3J and 3K**). Altogether, these oenocyte rescue data are consistent with *RhoBAD-PS* driving increased EGF signaling, potentially via non-C1-SOP cells.

### Increasing Sens affinity results in loss of segment-specific activation in abdominal SOPs

**Fig 4:**
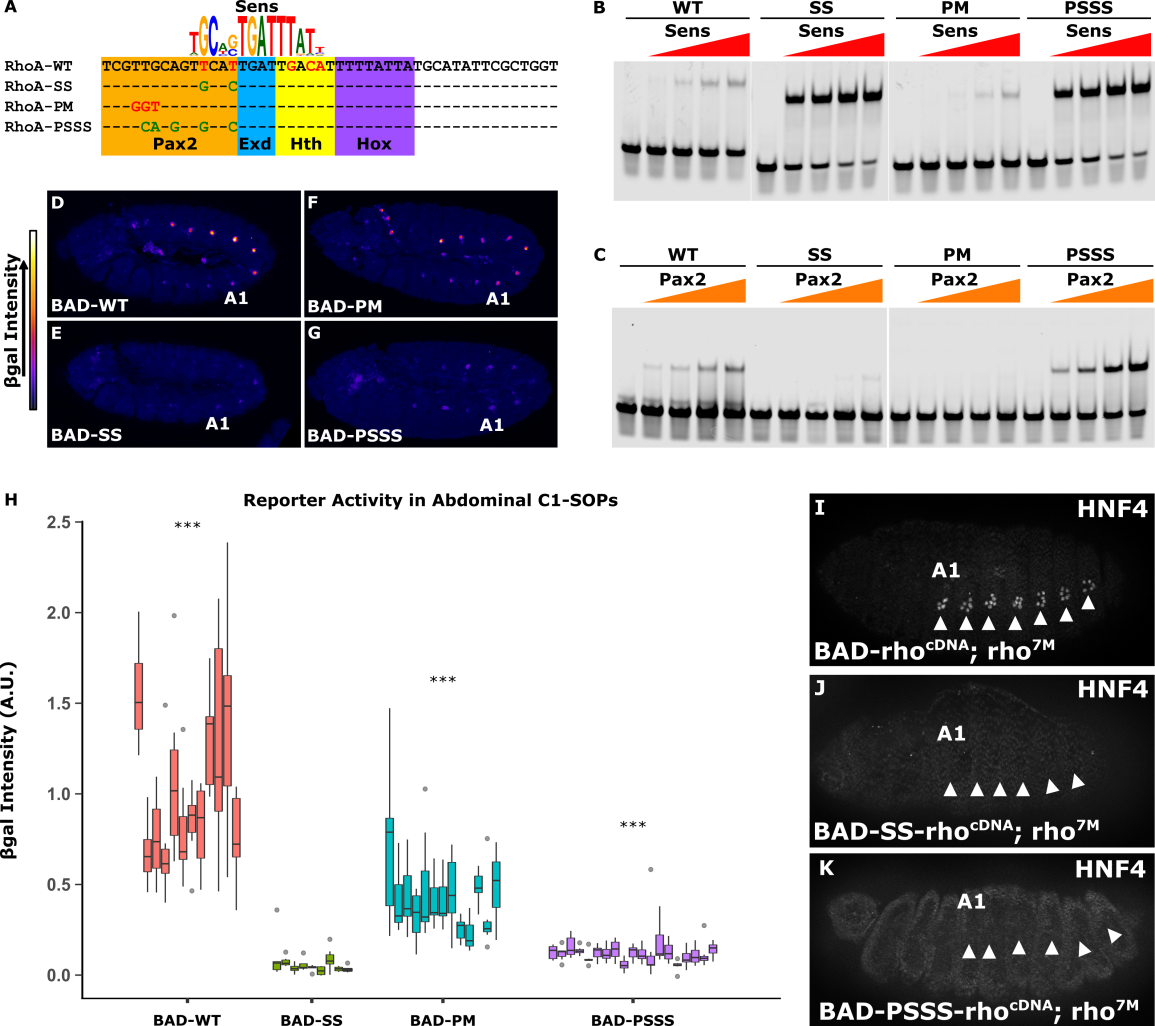
A high affinity Sens site results in repression of *RhoA* in abdominal C1-SOPs. **(A)** The SELEX-seq [23] Sens logo aligned with *RhoA* variants. Mis-matches are in red font, and sequence variants that improve the match are in green font. The Pax2, Exd, Hth, and Hox TFBSs are highlighted. **(B, C)** EMSAs using the indicated *RhoA* probes with either purified Sens (0, 23.5, 57, 114, and 228 ng) or Pax2 (0, 10.25, 20.5, 41, and 82 ng). Full gels are shown in **S3 Fig**. **(D-G)** Lateral view of stage 11 *RhoBAD-lacZ* (D), *RhoBAD-SS-lacZ* (E), *RhoBAD-PM-lacZ* (F), and *RhoBAD-PSSS-lacZ* (G) embryos immunostained for β-gal. β-gal intensity is represented by a heat-map at left. “A1” indicates the first abdominal segment. **(H)** Quantification of β-gal intensity in abdominal C1-SOPs in age-matched embryos. Each box represents measurements from a single embryo. *RhoBAD-SS-lacZ*, *RhoBAD-PM-lacZ*, and *RhoBAD-PSSS-lacZ* embryos were processed and imaged separately, each with *RhoBAD-lacZ* control embryos. Quantification for a representative set of *RhoBAD-lacZ* embryos are shown. β-gal intensities for each variant are reported as relative to the average β-gal intensity of control embryos. Two-tailed Welch’s T-test with Bonferroni correction was done to compare β-gal intensities to *RhobAD-SS* (* p < 0.05, ** p < 0.001, *** p < 0.0001), n = 12 (WT), 9 (SS), 13 (PM), and 19 (PSSS). (**I-K**) Lateral view of *RhoBAD-rho*^*cDNA*^ (I), *RhoBAD-SS-rho*^*cDNA*^ (J), and *RhoBAD-PSSS-rho*^*cDNA*^ embryos in a *rho*^7M^ background (stage 15) immunostained for an oenocyte marker (HNF4). Note, at least 10 embryos with transgenes containing high affinity Sens sites were analyzed and no oenocytes were observed.

Previous studies have found that increasing Sens TFBS affinity (*RhoA-SS*, **Fig 4A**) is sufficient to decrease abdominal *RhoBAD* activity [17] (**Fig 4E**). However, this experiment was performed prior to the discovery of an overlapping Pax2 site [22], and EMSAs reveal *RhoA-SS* not only increases Sens binding but also decreases Pax2 binding (**Fig 4B and 4C**). Hence, loss of *RhoBAD-SS* activity could be due to either increased repressor binding (Sens) or decreased activator binding (Pax2). To distinguish between these possibilities, we first compared the activity of *RhoBAD-PM* (which decreases Pax2 binding while leaving Sens binding) to the activity of *RhoBAD-SS* (which decreases Pax2 binding while simultaneously increasing Sens binding) (**Fig 4B and 4C**). Importantly, neither change substantially affects Exd/Hth/AbdA binding (**S3 Fig**). Comparative analysis of embryos with *RhoBAD-PM-lacZ* reveals a significant decrease, but not a complete loss, of abdominal C1-SOP activity; whereas *RhoBAD-SS-lacZ* embryos have a severe loss of activity in both abdominal and thoracic C1-SOPs (**Fig 4E, 4F and 4H** and **S5 and S6 Figs**). These results indicate that the lack of *RhoBAD-SS* activity is largely due to increased Sens binding, rather than loss of Pax2 binding. As a second test, we engineered a *RhoA* sequence with high affinity sites for both Sens and Pax2 (*RhoA-PSSS*). EMSAs reveal this sequence enhances Sens and Pax2 binding (**Fig 4B and 4C**) without affecting Exd/Hth/AbdA binding (**S3 Fig**). Reporter analysis demonstrates that *RhoBAD-PSSS-lacZ* embryos have nearly no activity in C1-SOP cells and behave much like *RhoBAD-SS-lacZ* (**Fig 4G and 4H** and **S7 Fig**). These results indicate that a strong Sens binding site can eliminate *RhoBAD* activity regardless of Pax2 affinity. Moreover, we found that *RhoBAD-SS-rho*^*cDNA*^ and *RhoBAD-PSSS-rho*^*cDNA*^ transgenes failed to rescue oenocyte development in *rho* mutant embryos (**Fig 4J and 4K**). By comparison, the wildtype *RhoBAD-rho*^*cDNA*^ transgene significantly induced oenoctye formation in abdominal segments (**Figs 3I and 4I**). Altogether, these experiments demonstrate that segment-specific *RhoBAD* activity requires a low affinity Sens TFBS.

### Overlapping activator and repressor sites are necessary for abdominal *RhoBAD* specificity

We previously found that abdomen-specific activity of *RhoA* is due to TF competition between a repressor (Sens) and activators (Pax2 plus Exd/Hth/Abd-A) [17, 22]. In this model, Exd/Hth/Abd-A and Pax2 bind *RhoA* in abdominal C1-SOP cells to both activate transcription and limit the binding of the Sens repressor (**Fig 1C and 1D**). Thoracic segments lack Abd-A expression, allowing Sens to bind and repress *RhoBAD* activity in the thorax. Moreover, the data in **Fig 4**suggest that competition between activators and repressors is a key feature of regulating output, as raising Sens affinity results in dominant repressor binding and a loss of *RhoBAD* activity in abdominal SOPs.

**Fig 5:**
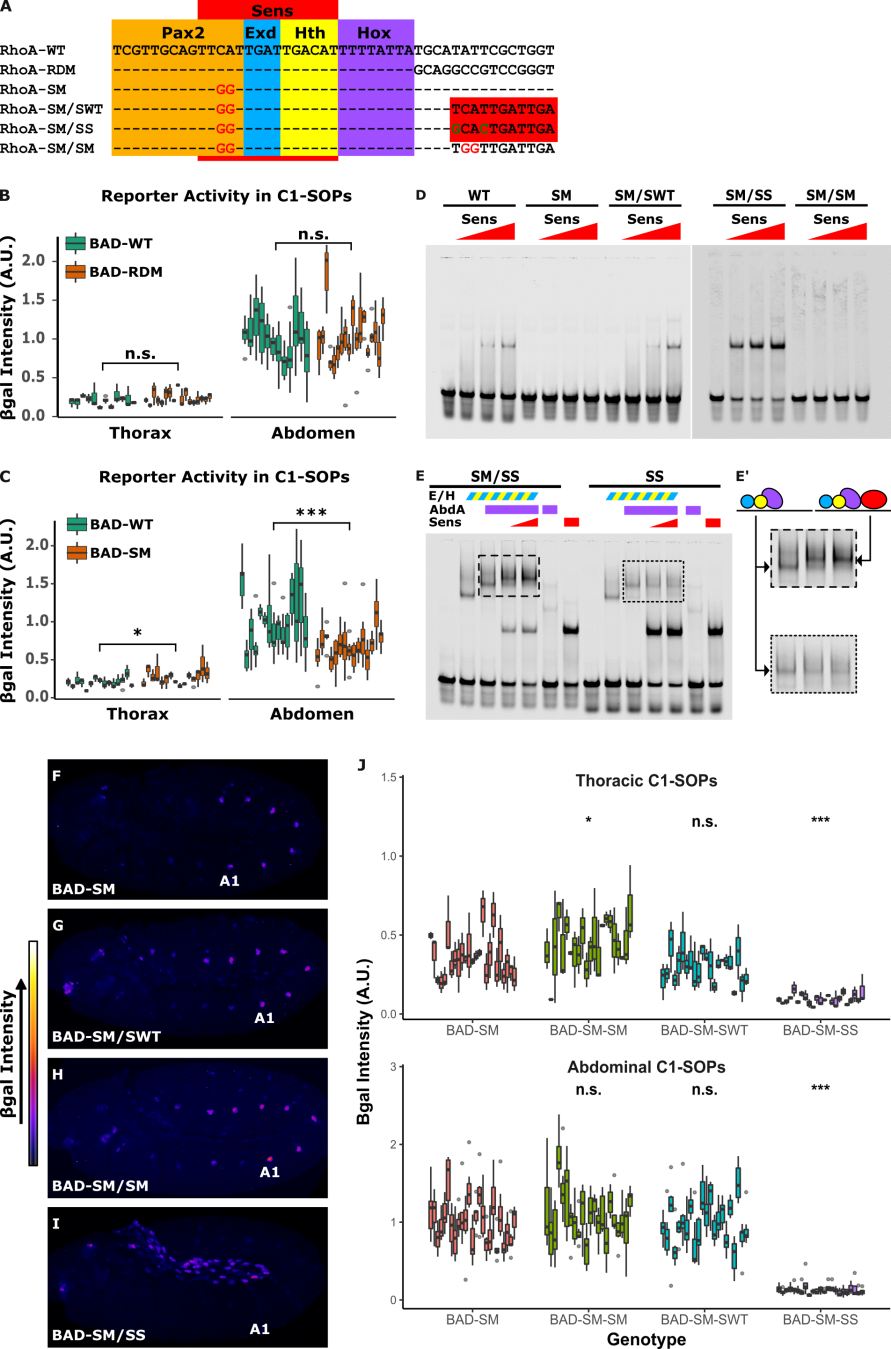
Overlapping activator and repressor binding sites are required for abdomen-specific *RhoA* activity. **(A)** Sequences of tested *RhoA* variants. *RhoA-RDM* contains random nucleotides downstream of the Hox site. *RhoA-SM* contains mutations that decrease Sens binding, and *RhoA-SM/SWT*, *RhoA-SM/SS* and *RhoA-SM/SM* add either a low affinity (WT), high affinity (SS), or mutant (SM) Sens site downstream of the Hox site. **(B-C)** Quantification of β-gal immunostaining intensities in C1-SOPs in *RhoBAD-LacZ versus RhoBAD-RDM-LacZ* (B) or *RhoBAD-SM-LacZ* (C). Each box summarizes measurements from a single embryo. Two-tailed Welch’s T-test was used to compare *RhoBAD-SM* and *RDM* mutants to wildtype, n = 12 (WT) and 18 (RDM) in (B) and n = 12 (WT) and 15 (SM) in (C). **(D)** EMSAs comparing binding of purified Sens to *RhoA* probes (0, 57, 114, and 228 ng of Sens). **(E)** EMSAs assessing competition between purified Sens (114 or 228 ng) against purified AbdA (189 ng) and Exd/Hth (59.2 ng) on *RhoA-SS* and *RhoA-SM-SS*. **(E’)** Close-up view of Exd/Hth/Hox and Exd/Hth/Hox/Sens complexes on DNA probes. Schematics denote the formation of each transcription factor complex. **(F-I**) Lateral view of stage 11 *RhoBAD-SM-lacZ* (F), *RhoBAD-SM/SWT-lacZ* (G), *RhoBAD-SM/SM-lacZ* (H), and *RhoBAD-SM/SS-lacZ* (I) embryos immunostained for β-gal. Intensity of β-gal stain is represented by heat-map at left. “A1” indicates first abdominal segment. Note, no *RhoBAD-SM/SS* activity is detected in the PNS and the activity that is observed is in cells of the gut. **(J)** Quantification of β-gal intensities in thoracic and abdominal C1-SOPs of noted *RhoBAD-lacZ* embryos. Each box represents measurements from a single embryo. Statistical analysis was done using Kruskal-Wallis test followed by post-hoc pairwise Mann-Whitney U test with Bonferroni correction, n = 25 (SM), 23 (SM/SM), 22 (SM/SWT), and 24 (SM/SS). For all statistical comparisons, n.s. p ≥ 0.05; * p < 0.05, ** p < 0.001, *** p < 0.0001.

To better understand the role of TFBS competition in segment-specific output, we created constructs that uncouple the repressor and activator TFBSs. To do so, we first tested a reporter with the sequences 3' to the Hox site randomly mutated (*RhoA-RDM*) and found it had similar activity as wild type *RhoBAD-lacZ* (**Fig 5A and 5B** and **S8 Fig**). Hence, this region can be altered without compromising *RhoBAD* activity. Next, we created a *RhoA* mutation that abolishes Sens binding (*RhoA-SM*) (**Fig 5A and 5D**) without altering activator binding (**S3 Fig**) [17]. Comparative analysis of *RhoBAD-lacZ* with *RhoBAD-SM-lacZ* revealed two expected results: First, loss of Sens binding resulted in a small but significant increase in thoracic expression compared to wild type *RhoBAD-lacZ* (**Fig 5C** and **S9 Fig**). Second, loss of Sens binding was not sufficient to equilibrate the C1-SOP levels between thoracic and abdominal segments. The latter result is consistent with thoracic segments lacking Abd-A, which plays an active role in stimulating transcription [22]. Unexpectedly, however, the Sens mutation also led to a small, but significant loss in abdominal SOP activity through mechanisms that are currently unclear (**Fig 5C**). Nevertheless, *RhoA-SM* eliminates Sens binding *in vitro* and alters *RhoBAD* activity *in vivo*, and thereby provides a platform for uncoupling the activator and repressor sites.

To generate sequences that lack TF binding competition, we created *RhoA* variants that lack an endogenous Sens site (*RhoA-SM*) and provide a new Sens site downstream of the Hox site. Three variants were tested: (1) a mutant Sens site (*RhoA-SM/SM*); (2) the wild type low affinity site (*RhoA-SM/SWT*); and (3) a high affinity Sens site (*RhoA-SM/SS*) (**Fig 5A**). EMSAs reveal that while Sens fails to bind *RhoA-SM* and *RhoA-SM/SM*, it binds the re-engineered *RhoA-SM/SWT* similarly to *RhoA-WT* and binds *RhoA-SM/SS* with greater affinity (**Fig 5D**). Importantly, moving the Sens site is sufficient to permit co-binding of activator and repressor TFs *in vitro* as Sens and Exd/Hth/Abd-A proteins simultaneously bind *RhoA-SM/SS* but not *RhoA-SS*, which has overlapping binding sites (**Fig 5E**).

Comparative analysis of the *RhoBAD* variants revealed that competition for overlapping TFBSs is essential for proper output (**Fig 5F–5J** and **S10 Fig**). First, we found that the re-engineered wild-type Sens site (*RhoBAD-SM/SWT*) is insufficient to repress reporter activity and behaves similarly as *RhoBAD-SM* and *RhoBAD-SM/SM*, which both lack Sens binding (**Fig 5F–5H and 5J**). Hence, a low affinity Sens site that is uncoupled from the activator sites (Pax2/Exd/Hth/Hox) is unable to repress either abdominal or thoracic SOP activity. In sharp contrast, the re-engineered high-affinity Sens site (*RhoBAD-SM/SS*) results in gene repression in both thoracic and abdominal SOPs (**Fig 5I and 5J**). This finding suggests that Sens can inhibit *RhoBAD* activation through mechanisms other than sterically blocking the binding of activator TFs. Moreover, these findings are consistent with the hypothesis that low affinity Sens sites are required to allow the abdominal Hox and Pax2 activators to stimulate *RhoBAD* expression. Hence, two features of *RhoA* are critical to yield segment-specific *RhoBAD* activity in abdominal C1-SOP cells: 1) low affinity Sens and Pax2 sites are required, and 2) the TFBSs overlap to ensure independent binding of activator versus repressor complexes.

### Inverse correlation between PWM information content and accuracy

**Fig 6:**
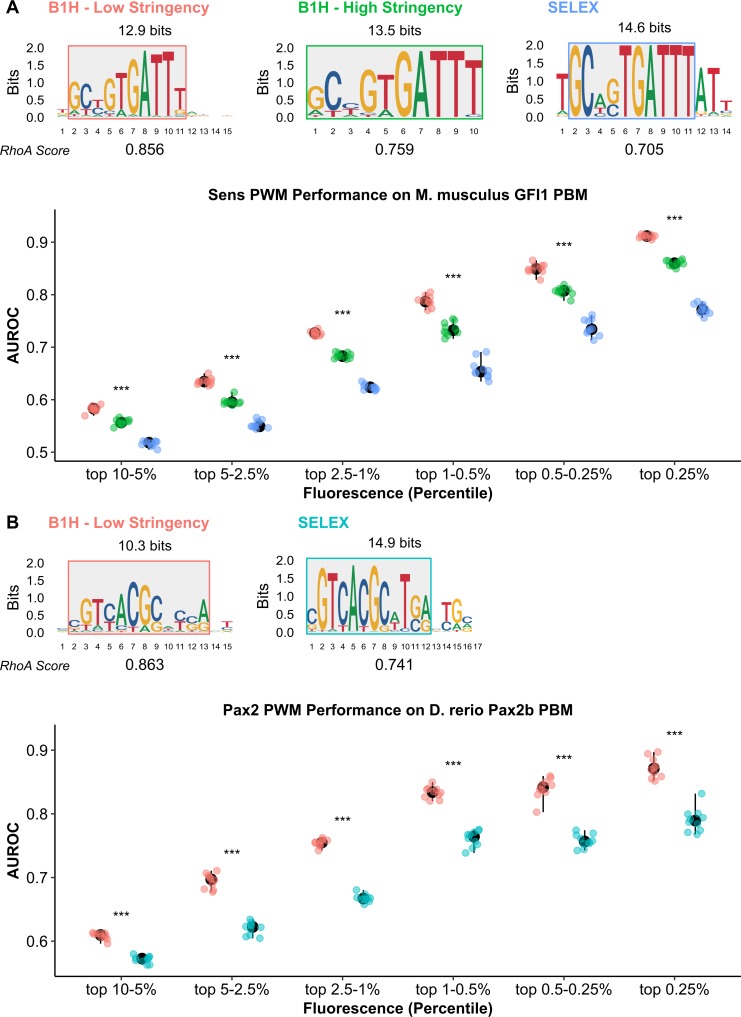
Inverse correlation between PWM information content and the ability to identify low-affinity Pax2 and Sens TFBSs. **(A, B)** Top Panel: Published Sens and Pax2 PWMs placed in order from lowest to highest information content (left-to-right). PWMs were derived from published B1H (low and high stringency) and SELEX-seq assays [23, 24]. Flanking low information positions were removed to make all PWMs the same length (shaded boxes). Total information content (bits) of the trimmed PWMs is indicated above each PWM. The Relative Log-Likelihood (RLL) score of each PWM on *RhoA* is indicated below the PWMs. Bottom Panel: AUROC of each published PWM for discriminating PBM probes (binned by fluorescence, as indicated on x-axis) from 10 sets of non-specific probes (matched number of control probes randomly selected from the 50% of probes with the lowest fluorescence). Sens and Pax2 PWMs were tested on PBMs for the vertebrate homologs *H*. *sapiens* Gfi1b and *D*. *rerio* Pax2b. Statistical comparisons were conducted using Kruskal-Wallis test. P-values were Bonferroni-adjusted to correct for multiple comparisons (* p < 0.05, ** p < 0.01, *** p < 0.001).

Since CRM studies have increasingly found that low affinity sites are required for accurate output, we next assessed the utility of PWMs to predict such sites for Sens and Pax2. As described above, PWMs derived from SELEX-seq [23] assays for Sens and Pax2 failed to score *RhoA* above a 0.8 RLL threshold (**Fig 2A**). Additional published PWMs derived from bacterial-1-hybrid (B1H) assays (FlyFactorSurvey project) are available for Sens and Pax2 [24]. In B1H assays, “hits” are selected when a TF binds a sequence and activates survival gene expression in the presence of an inhibitor, such that increasing inhibitor concentrations select for higher affinity TFBSs [24]. For Sens, B1H assays were previously performed under “high" and “low” stringency conditions, whereas a single Pax2 B1H assay was conducted under low stringency (**Fig 6A**, top panel) [24]. Comparing the B1H and SELEX-seq PWMs revealed similar motifs, but with differences in their degree of degeneracy (**Fig 6A and 6B**, top panels). “Degeneracy” can be defined as the inverse of information content, which is measured in bits and represented by letter height in a sequence logo. We used each PWM to score *RhoA* and found that only the "low" stringency B1H derived PWMs successfully scored the Pax2 and Sens sites above the 0.8 RLL threshold (**Fig 6A and 6B**, top panel).

To more broadly assess the ability of each PWM to predict both high and low affinity sites, we analyzed published protein-binding microarray (PBM) data for vertebrate homologs of Pax2 and Sens (*Danio rerio* Pax2b and *Homo sapiens* Gfi1b) [29, 30]. These homologs share 88% and 85% sequence identity with the Pax2 and Sens binding domains, respectively; and thus, are likely good models for Pax2 and Sens binding specificities [23, 29]. A key advantage of PBM assays is binding strength (as measured by fluorescence intensity) positively correlates with binding affinity, thereby permitting scoring probes across a range of affinities. We scored bound probes of different fluorescent intensities using the B1H and SELEX PWMs, and used the Area-Under-the-Receiver-Operating-Characteristic (AUROC) to measure the ability of each PWM to discriminate bound probes (binned by fluorescence intensity) from non-specific sequences. For non-specific sequences, we randomly selected a matched number of probes from the bottom 50% fluorescence. Note, when AUROC values approach 0.5, the PWMs no longer reliably distinguish bound probes from non-specific sequences. This assessment surprisingly shows that the more degenerate PWMs (“B1H Low Stringency”) are not only more accurate in identifying low-affinity probes, but are also significantly more accurate in identifying high-affinity probes (**Fig 6A and 6B,** bottom panels).

**Fig 7:**
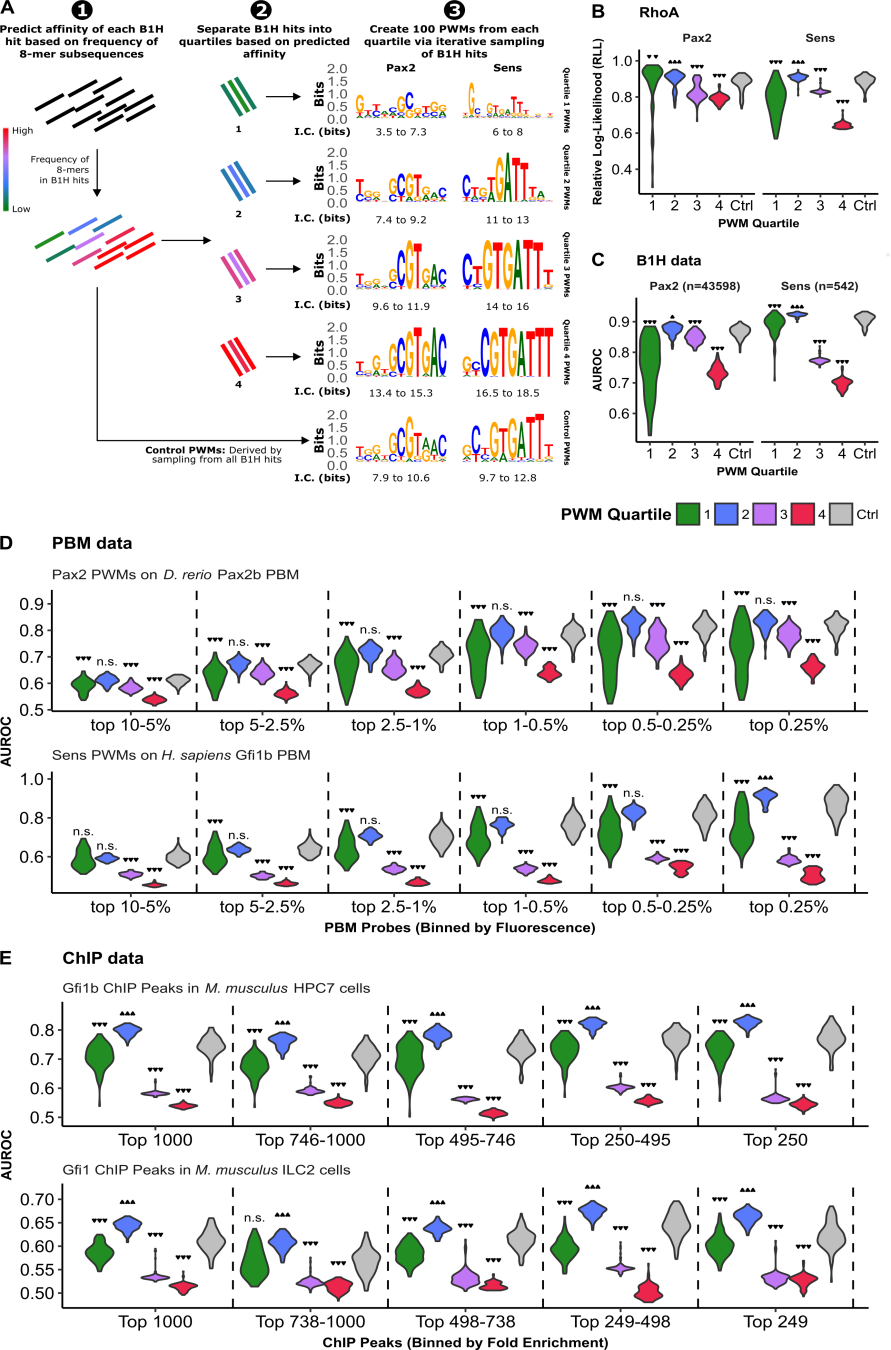
High information content PWMs are less accurate at identifying TFBSs obtained from both *in vitro* and *in vivo* binding events. **(A)** Schematic describing how PWMs were created by sub-sampling Sens and Pax2 B1H hits. Each B1H hit was placed into quartiles based on 8-mer sequence frequency within the pool of B1H hits. 100 PWMs were generated by iteratively sampling 50 B1H hits from each quartile. 100 PWMs were also generated by sampling 50 B1H hits from the entire pool (Control PWMs). The range of total information content (I.C.) for PWMs in each quartile are indicated below the motifs. **(B)** Relative log-likelihood (RLL) score of each PWM for the *RhoA* sequence. **(C)** AUROC of each PWM for discriminating low-stringency B1H hits from shuffled sequences. **(D)** AUROC of each PWM for discriminating bound PBM probes (binned by fluorescence, as indicated on x-axis) from non-specifically bound probes (matched number of control probes randomly selected from the 50% of probes with the lowest fluorescence). **(E)** AUROC of each Sens PWM for discriminating *M*. *musculus* Gfi1 and Gfi1b ChIP-seq peaks from random, non-repetitive genomic sequences. Gfi1b ChIP-seq was conducted using multipotent Hematopoietic Progenitor cells (HPC-7) and Gfi1 ChIP was conducted using innate Type-2 Lymphocytes (ILC2) [32, 33]. Analysis was limited to the 1000 peaks with greatest fold enrichment per ChIP dataset, and ChIP peaks were binned by fold enrichment as indicated on x-axis. For panels **C**-**E**, AUROCs represent the median using 10 different sets of negative sequences. All violin plots are scaled to have the same width. Statistical analysis was performed using Kurskal-Wallis test followed by a post-hoc pairwise Mann-Whitney U test. P-values were Bonferroni-adjusted due to multiple comparisons arising from groups of PWMs (all panels) and binning of sequences (panels **D** and **E**) (n.s. p ≥ 0.05; * p < 0.05; ** p < 0.01, *** p < 0.001).

The above findings suggest that DNA libraries that include low affinity TFBSs (i.e. B1H low stringency assays) produce lower information content PWMs with increased accuracy. To more thoroughly assess how the affinity of binding sites used to generate PWMs affects TFBS accuracy, we developed a method to sub-group B1H hits based on predicted affinity and compare the performance of PWMs generated from each sub-group (**Fig 7**). For this analysis, we hypothesized that B1H hits containing highly represented sequences are more likely to contain high affinity sites. Indeed, the number of times an 8-mer appears among all B1H hits correlates with the 8-mer E-scores derived from PBMs for related homologues (Spearman’s rank correlation >0.8) (**S11 Fig**). Therefore, we divided the 542 B1H hits for Sens and the 43,112 B1H hits for Pax2 into quartiles based on 8-mer frequency and derived 100 PWMs from each quartile by iteratively sampling 50 B1H hits from each quartile followed by the MEME algorithm [31] to generate PWMs (**Fig 7A**, see **Methods** for details). In such a manner, B1H hits within higher quartiles contain more highly represented sequences, and thus, generate PWMs with greater information content (**Figs 7A and S12**). As a control, we created a set of 100 PWMs by iterative sampling 50 B1H sequences from the unfiltered dataset (“control PWMs”). To determine how well PWMs from each quartile predict TFBSs compared to the control PWMs, we assessed their ability to score *RhoA* (**Fig 7B**), discriminate B1H hits from shuffled sequences (**Fig 7C**), and score PBM probes bound by the vertebrate Gfi1b and Pax2 factors (**Fig 7D**). We found that relatively low-information content PWMs from Quartile 2 performed significantly better than the control PWMs when scoring the *RhoA* sequence (**Fig 7B**). In contrast, PWMs generated using highly represented sequences (Quartiles 3 and 4) scored *RhoA* significantly worse than Quartile 2 and control PWMs. Moreover, a similar trend was found when assessing the accuracy of PWMs to discriminate B1H hits from shuffled sequences (**Fig 7C**) or when scoring PBM probes binned based on fluorescent intensity (**Fig 7D**). For example, Quartile 3 and 4 PWMs for both Pax2 and Sens have lower AUROC values for discriminating B1H hits from shuffled sequences and for discriminating even the most highly bound PBM probes compared to lower information content PWMs derived from either Quartile 2 or control PWMs (**Fig 7C and 7D**). It should be noted, however, that the very low information content PWMs from Quartile 1 do not perform as well, as evidenced by wider variance for *RhoA* score predictions (**Fig 7B**) and significantly lower AUROC values (**Fig 7C and 7D**).

Lastly, we investigated the ability of PWMs from each quartile to predict DNA binding events in cells using ChIP-seq data. While high quality ChIP-seq data are not available for Pax2 in either vertebrates or *Drosophila*, several published ChIP-seq experiments have been conducted for Gfi1 and Gfi1b in mammalian cells [32–40]. Using 10 published *H*. *sapiens* and *M*. *musculus* Gfi1 and Gfi1b ChIP-seq datasets, we assessed the ability of Sens PWMs to discriminate ChIP-seq peak sequences from an equal number of random, non-repetitive genomic DNA sequences. Initially we analyzed all called ChIP-seq peaks in each dataset and found that Quartile 2 PWMs out-performed all other PWMs in 7 of the 10 ChIP datasets, whereas all of the PWMs performed poorly (AUROC close to 0.5) on the remaining 3 ChIP datasets (**S13 Fig**). We next asked if the PWMs derived from higher affinity B1H hits (Quartile 3 and 4 PWMs) would perform better when analyzing only the strongest ChIP peaks. Therefore, we binned the top one thousand ChIP peaks from two representative ChIP-seq datasets [32, 33] based on fold enrichment and analyzed the bins separately. As expected, there is a general trend for all the PWMs to perform better as fold enrichment of ChIP peaks increases (**Fig 7E**). Interestingly, Quartile 2 PWMs out-performed almost all other PWMs in predicting ChIP peaks, regardless of fold enrichment (**Fig 7E**). Moreover, PWMs derived from the high-affinity B1H hits (Quartile 3 and 4 PWMs) had significantly less discriminatory power, even when predicting the most highly enriched ChIP-peaks. Thus, these findings suggest that, at least for Sens/Gfi1, using more degenerate PWMs derived from lower affinity sites better predicts TFBSs from both *in vitro* (B1H and PBM assays) and *in vivo* DNA binding data (*RhoA* and ChIP-seq).

## Discussion

In this study, we used quantitative DNA binding and transgenic assays to interrogate how Sens and Pax2 TFBSs contribute to cell- and segment-specific CRM activity and thereby EGF signaling in the *Drosophila* embryo. Our findings reveal that *RhoA* requires overlapping low-affinity TFBSs to accurately regulate transcription in abdominal SOP cells. In addition, we performed a computational analysis to interrogate the effectiveness of different PWMs for distinguishing TFBSs from background sequences. Taking the B1H, PBM, and ChIP-seq analysis together, our results demonstrate that low-information content PWMs better identify Sens and Pax2 TFBSs. Overall, these findings have important implications for two areas of biology: How CRM composition contributes to transcriptional outcome, and the properties of PWMs that best predict biologically meaningful TFBSs.

### CRM design principles: The role of low affinity and overlapping TFBSs

CRMs consist of TFBSs that integrate numerous inputs to determine transcriptional output. Three primary models for how CRMs regulate expression have been proposed [3] (A) the “Flexible Billboard” model posits that each TFBS independently recruits a TF that contributes to transcription in an additive manner, and thus the arrangement of TFBSs is of little importance [41]. (B) The “TF Collective” model posits that TFs work cooperatively, but that protein-protein interactions between TFs allow for flexible TFBS arrangement [42, 43]. (C) The “Enhanceosome” model posits that TFs form cooperative complexes that are constrained by the arrangement of TFBSs [44, 45]. While a few CRMs have been categorized according to these models, it is currently unclear what proportion of CRMs each of these models represent.

Our study reveals that the arrangement of TFBSs is important for the activity of the *RhoBAD* CRM. However, unlike the enhanceosome, which is constrained by cooperative TF complex formation, *RhoBAD* is instead constrained by competition for overlapping TFBSs. Hence, uncoupling repressor and activator TFBSs (*RhoA-SM/SWT*) in *Drosophila melanogaster* results in abnormal activity. This finding is consistent with a mechanism of steric exclusion, which thereby constrains the locations of the TFBSs. In fact, comparative analysis of the *RhoA* sequence across numerous *Drosophilid* species suggests that low affinity and overlapping Sens and Pax2 TFBSs are a conserved feature of *rhomboid* regulation (**S14 Fig**). Moreover, we found that the affinity of the overlapping sites is tuned to yield appropriate cell- and segment-specific outputs. Specifically, we show that enhancing Sens affinity to *RhoBAD* results in the loss of activation in abdominal segments, whereas increasing Pax2 affinity increases activation in a subset of ectopic PNS cells. In this way, *RhoBAD* combines features of two previously studied CRMs: the *sparkling* enhancer, which requires low affinity Suppressor of Hairless (Su(H)) sites for cell-specific expression in the *Drosophila* eye [46], and the *shavenbaby* enhancer that requires low affinity Hox sites to generate segment-specific outcomes in the *Drosophila* abdomen [5]. Hence, the overlap of low affinity sites for TFs expressed in the PNS (Sens and Pax2) and the abdomen (Abd-A) yields both cell- and segment-specific *RhoBAD* activity.

Our studies also have implications for how TFBS affinity can affect the mechanism used by a TF to ensure appropriate outputs. For example, Sens can repress *RhoBAD* activity via a high affinity TFBS, even if it does not overlap the activator sites. This finding indicates Sens uses a repressive activity that is not solely dependent on steric exclusion of activators. These results are consistent with studies demonstrating that the mammalian Sens homologues (Gfi1/Gfi1b) recruit repressive chromatin remodelers, such as HDAC-1 [47]. Moreover, Sens-mediated repression is dominant over a strong Pax2 TFBS, as demonstrated by the lack of activity of *RhoBAD-PSSS* (**Fig 4**). Hence, a low affinity Sens site and overlap with activator TFBSs are both required for proper CRM output, suggesting that these requirements constrain the ability of *RhoBAD* to tolerate sequence changes.

### Building PWMs that accurately predict TFBSs from complex datasets

A common approach to predict functional TFBSs within CRMs has been to use large-scale *in vitro* DNA binding data from assays such as B1H, SELEX-seq, or PBM to create models of TF binding specificity [23, 24, 29, 48–50]. In addition, *in vivo* approaches, such as ChIP-seq or DamID assays, have been increasingly used to create PWMs from cells and tissues [40, 51]. Hence, for many TFs, several PWMs have been generated using data from different methods (see the CIS-BP database [29]); and for the biologist that wants to predict TFBSs, this raises the issue of which PWMs are best suited to identify functional TFBSs from genomic datasets?

Different PWMs for a given TF typically share common core sequences, but often vary in information content (i.e. degeneracy). A recent study compared a variety of algorithms to generate PWMs and found that in general those with lower information content performed better in predicting TFBSs [50]. Consistent with these results, we found an inverse relationship between PWM information content and accuracy for Sens and Pax2 –TFs from two distinct families (C2H2 Zinc Finger and Paired-Box TFs, respectively). Moreover, this relationship was observed with both published PWMs (**Fig 6**) as well as by selective sampling of B1H hits to create hundreds of PWMs (**Fig 7**). For example, by systematically comparing Sens PWMs generated using TFBSs of different predicted affinities, we determined that eliminating high affinity sites resulted in PWMs with increased predictive accuracy for both *in vitro* (B1H and PBM) and *in vivo* (*RhoA* and mammalian ChIP-seq data) DNA binding events. In contrast, using only B1H hits predicted to be of high-affinity resulted in over-representation of certain sequence motifs and, consequently, high-information content PWMs with poor accuracy. Thus, regardless of which DNA binding assay (SELEX, B1H, or PBM) is used to generate a library of sequences, care must be taken to ensure the library is sufficiently diverse to create PWMs that can accurately identify both low and high-affinity TFBSs. However, our approach also highlights that increasing PWM degeneracy has its limits, as highly degenerate PWMs created using the least-represented sequences (Quartile 1, **Fig 7**) resulted in highly erratic predictions. This finding may be due to the least represented sequences containing rare binding events and/or false positive sequences.

While TFs can interact with the genome over a range of affinities [52, 53] and CRMs with low-affinity sites have been identified [5–9, 45, 54–56], the prevalence of low-affinity interactions between TFs and DNA remains unclear. While our study does not definitively address this question, our analysis of Gfi1 and Gfi1b (mammalian Sens homologues) *in vivo* binding found that the same low information content PWMs that best discriminated *RhoA*, B1H hits, and PBM data from random sequences also performed significantly better at identifying potential TFBSs within ChIP-seq peaks (**Fig 7**). It should be noted that a consequence of degenerate DNA binding is that the number of high-affinity TFBSs within a genome are likely to be greatly outnumbered by the number of low affinity sequences in the genome. Moreover, protein-protein interactions between TFs can modify binding preferences [48]. Therefore, less restrictive models of TF binding may have greater accuracy for identifying TFBSs within ChIP-seq peaks because low affinity sites and modified binding preferences are less penalized than they are by more restrictive models of TF binding.

## Materials and methods

### Generation of transgenic fly lines

*RhoBAD* mutations (see sequences in Figures) were created using site-directed mutagenesis–primers available on request. Each mutation was cloned into *pLacZattB*, confirmed by DNA sequencing, and integrated into the same genomic location (51C) using ΦC31 (Rainbow Transgenics Inc.).

### Embryo immunostaining

For quantitative expression analysis, all embryos were harvested, fixed, immunostained, and imaged under identical conditions. Each variant transgene was compared directly with an appropriate control: *RhoBAD-lacZ* or *RhoBAD-SM-lacZ* (in **Fig 5J**). The primary antibodies used were: Abd-A (Guinea Pig 1:500, [17]), Sens (Rat 1:125, [57]), β-gal (Chicken 1:1000, Abcam), and Pax2 (Rabbit 1:2000, [58]). Secondary antibodies conjugated to Alexa-Fluor molecules were purchased from Molecular Probes. Imaging was performed using a Nikon A1 LUNA inverted confocal microscope. Z-Stacks were mean-projected using Fiji (Bioformats plug-in to read ND2 files) [59–61]. NIS-Elements software was used to segment and quantify β-gal intensities in C1-SOPs. Raw measurements used to create graphs are provided in **S6 Data**.

### Protein purification, EMSAs, and predicted binding calculations

His-tagged proteins were purified from BL21 cells using Ni-chromatography as previously described [62]: Abd-A [63]; Exd/Hth heterodimers [62]; Sens [17]; and Pax2 [22]. Proteins were confirmed using SDS-PAGE and Coomassie staining and concentrations measured by a Bradford assay. EMSAs were performed using native polyacrylamide gel electrophoresis [43, 64]. Probes were used at 0.36 μM, and protein concentrations were noted in figure legends. Acrylamide gels were imaged using the LICOR Odyssey CLx scanner and densitometry was performed using ImageJ. All quantitative EMSAs were done in triplicate. Predicted binding (**Fig 2C** and **2F**) was calculated as follows:

$$Predicted\;Binding=\frac{M}{1+e^{E-\mu }}$$ (1)

*E* is the PWM Energy Score for the site; *μ* is the chemical potential, which was derived by fitting to data using gradient descent (0.176 for Pax2 and 1.03 for Sens); and *M* is a scaling factor (equal to the maximum observed probe bound) [25–27].

### Creation of PWMs

Sens and Pax2 PWMs were derived from: (1) B1H PWMs were downloaded from the FlyFactorSurvey website (http://mccb.umassmed.edu/ffs/) [24]; (2) SELEX-seq PWMs were downloaded as position count matrices (PCMs) from Nitta *et al*. [23] (**S1 Data**). PCMs were converted to PWMs using a custom R script using a pseudo-count of $\sqrt{n}$ (where *n* is the number of observed nucleotides at a position). Sequence logos were created using the ggSeqLogo package for R [65].

To generate PWMs in **Fig 7**, B1H hits were (1) assigned an affinity score (defined below), (2) placed into quartiles, (3) 50 B1H hits were sampled from each quartile and (4) MEME was used to generate PWMs using the following parameters: *-dna -nmotifs 1 –revcomp -mod oops* [31]. These parameters indicate that (a) a DNA sequence has been inputted into MEME, (b) a single motif should be found, (c) the reverse complement is analyzed, and (d) the motif occurs only once per sequence. Steps 3 and 4 were repeated 100 times to generate 100 PWMs from each quartile. FlyFactorSurvey B1H hits are 25-mer (Pax2) or 27-mer (Sens) sequences. To calculate a predicted affinity score, each B1H hit was separated into un-gapped 8-mers and the number of occurrences of each 8-mer in the total pool of B1H hits was determined; the predicted affinity score is equal to the maximum occurrence of all 8-mers composing a B1H hit.

### Scoring sequences with PWMs

Using custom R scripts, we scored *RhoA*, B1H, PBM, and ChIP-seq sequences using the relative log-likelihood method [66], as follows:

$$Relative\;Log\;Likelihood=\frac{x-S_{min}}{S_{max}-S_{min}}$$

$$\begin{array}{c} x=log‑likelihood\;score\\ S_{\min}=minimum\;possible\;score\\ S_{\max}=maximum\;possible\;score \end{array}$$ (2)

For all sequences, a sliding window was used to score the forward and reverse strands, and the score assigned is equal to the highest score produced. To allow partial matches to the PWM on the edges of sequence, two ambiguous nucleotides (i.e. “NN”) were added to both ends of each scored sequence. These ambiguous nucleotides receive a score of 0.

### Assessing PWMs

PWMs were assessed by their ability to discriminate known binding sites (score B1H hits, ChIP peaks, and high fluorescence PBM probes) from control sequences. This ability to discriminate was measured by the Area-Under-the-Receiver-Operating-Characteristic (AUROC)–a commonly used metric of the ability of a predictor (i.e. a PWM) to differentially score two sets of items (i.e. differentially score B1H hits, ChIP peaks, and high fluorescence PBM probes from control sequences). The more effectively a PWM scores can distinguish sequences containing binding sites from control sequences (regardless of the absolute score the PWM assigns to the sequences) the greater the AUROC value. In all instances, we assessed the AUROC using 10 sets of control sequences and assigned the median AUROC value to the PWM. Control sequences were the same number and length as the positive sequences.

B1H hits (“Unique Raw Sequence”) were downloaded from FlyFactorSurvey (http://mccb.umassmed.edu/ffs/) [24]. Control B1H sequences were created through mono-nucleotide shuffling.

*H*. *sapiens* and *M*. *musculus* Gfi1 and Gfi1b ChIP-seq peaks were downloaded from the NCBI GEO database as BED files [32–40]. Peaks were trimmed to the central 100 bp. For each ChIP dataset, control sequences were generated by randomly selecting an equal number of genomic loci that did not overlap with peaks in the ChIP dataset and did not overlap with UCSC Repeatmasker regions [67]. For two ChIP datasets (GSE50806 [32] and GSM552235 [33]) the same analysis was repeated using the top 1000 peaks, as defined by the MACS2 “fold enrichment” score [68] (**Fig 7**). Fold enrichment was calculated by downloading raw reads (SRA projects SRP029908 [32] and SRP002575 [33]); assessing read quality with FASTQC [69] (**S2–S5 Data**); mapping to the mm10 genome using Bowtie 2 [70]; and calling and scoring peaks using MACS2 [68].

PBM data were downloaded from CIS-BP (*D*. *rerio Pax2b*, M1499_1.02) and UniPROBE (*H*. *sapiens Gfi1b*, UP00592) [29, 30]. AUROC analysis was done on PBM probes binned by fluorescence (top 0.25%, top 0.5%-0.25%, top 1%-0.5%, top 2.5%-1%, top 5%-1%, top 10%-5%). For each bin, control sequences were generated by randomly selecting PBM probes in the bottom 50% of fluorescence.

### Data processing and plotting

Data processing was conducted in R using Bioconductor, Tidyverse, and AUC packages, and plotted using ggplot2, ggpubR, and gridExtra packages [71–77].

## Supporting information

S1 Fig The *RhoA* Exd, Hth, and AbdA binding sites match well with their corresponding PWMs. PWMs were downloaded from the FlyFactorSurvey website and aligned to the *RhoA* sequence. The RLL score for each *RhoA* transcription factor site is listed.
(TIF)

S2 Fig Comparative DNA binding analysis of Pax2 and Sens to *RhoA* and B1H-derived binding sites. EMSA analysis for the binding of Pax2 **(A-B)** and Sens **(C-D)** to wildtype *RhoA* probes or *RhoA* probes in which the Sens or Pax2 binding sites have been replaced with B1H hits. Sequence IDs correspond to sequences in **Fig 2A and 2D**. **(A, C**) EMSAs performed, respectively, with 48 ng of Pax2 or 106 ng of Sens in triplicate. These EMSAs were quantified to produce graphs in **Fig 2C and 2F**. **(B, D**) Uncropped versions of gels shown in **Fig 2B and 2E**.
(TIF)

S3 Fig Comparative DNA binding analysis of Pax2, Sens, and Exd/Hth/AbdA to *RhoA* variant sequences. EMSA analysis for the binding of Exd/Hth/Hox **(A)**, Sens **(B)**, or Pax2 **(C)** to probes carrying each *RhoA* variant shown in **Figs 3**and 4. The protein concentrations used in were as follows: Exd/Hth: 59.2 ng; AbdA: 94.5 and 189 ng; Sens: 0, 23.5, 57, 114, and 228 ng; Pax2: 0, 10.25, 20.5, 41, and 82 ng. These panels show the complete gels, whereas relevant portions of these gels are shown in **Figs 3**and 4.
(TIF)

S4 Fig Comparison of Sens levels of non-C1-SOPs in *RhoBAD-WT* and *RhoBAD-PS* embryos. Each boxplot represents Sens levels in non-C1 Sens+ nuclei per embryo. Note, no significant difference in Sens levels were observed between reporter genotypes. Statistical analysis was conducted using the Welch’s T-test to compare mean reporter activity per embryo between the two genotypes.
(TIF)

S5 Fig Comparison of AbdA, β-gal, and Sens levels in C1-SOPs of *RhoBAD-WT* and *RhoBAD-SS* embryos. Each boxplot represents the indicated AbdA, Sens, and β-gal levels in either thoracic or abdominal C1-SOPs. Note, no significant difference in Sens or AbdA levels were observed between reporter genotypes. Statistical analysis was conducted using the Welch’s T-test to compare mean reporter activity per embryo between the two genotypes.
(TIF)

S6 Fig Comparison of AbdA and β-gal levels in C1-SOPs of *BAD-WT* and *BAD-PM* embryos. Each boxplot represents the indicated AbdA and β-gal levels in either thoracic or abdominal C1-SOPs. Note, there is a significant difference in abdominal β-gal levels, but no significant difference in AbdA levels between reporter genotypes. Statistical analysis was conducted using the Welch’s T-test to compare mean reporter activity per embryo between the two genotypes.
(TIF)

S7 Fig Comparison of AbdA, β-gal, and Sens levels in C1-SOPs of *BAD-WT* and *BAD-PSSS* embryos. Each boxplot represents the indicated AbdA, Sens and β-gal levels in either thoracic or abdominal C1-SOPs. Note, there is a significant difference in abdominal β-gal levels, but no significant difference in AbdA or Sens levels were observed between reporter genotypes. Statistical analysis was conducted using the Welch’s T-test to compare mean reporter activity per embryo between the two genotypes.
(TIF)

S8 Fig Comparison of AbdA and β-gal levels in C1-SOPs of *BAD-WT* and *BAD-RDM* embryos. Each boxplot represents the indicated AbdA and β-gal levels in either thoracic or abdominal C1-SOPs. Note, no significant difference in levels were observed between reporter genotypes. Statistical analysis was conducted using the Welch’s T-test to compare mean reporter activity per embryo between the two genotypes.
(TIF)

S9 Fig Comparison of AbdA, β-gal, and Sens levels in C1-SOPs of *BAD-WT* and *BAD-SM* embryos. Each boxplot represents the indicated AbdA, Sens and β-gal levels in either thoracic or abdominal C1-SOPs. Note there is a significant difference in β-gal levels, but no significant difference in AbdA or Sens levels were observed between reporter genotypes. Statistical analysis was conducted using the Welch’s T-test to compare mean reporter activity per embryo between the two genotypes.
(TIF)

S10 Fig Comparison of AbdA, β-gal, and Sens levels in C1-SOPs of *BAD-SM*, *BAD-SM/SM*, *BAD-SM/SWT*, and *BAD-SM/SS* embryos. Each boxplot represents the indicated AbdA, Sens and β-gal levels in either thoracic or abdominal C1-SOPs. Note, although *BAD-SM/SM*, *BAD-SM/SWT*, and *BAD-SM/SS* have small, but statistically significant, differences in AbdA and Sens levels in C1-SOPs relative to *BAD-SM*; the only genotype to have substantial and statistically significant decrease in β-gal levels was *BAD-SM/SS*. Statistical analysis was conducted using the Kurskal-Wallis test followed by a post-hoc pairwise Mann-Whitney U test. P-values were Bonferroni adjusted for multiple comparisons.
(TIF)

S11 Fig Analysis of 8-mer occurrences within Bacterial-1-Hybrid (B1H) sequences and relationship to affinity. **(A, B)** Histograms displaying the distribution of 8-mer occurrence scores of B1H hits for Sens (FlyFactorSurvey ID Sens_SOLEXA_5) and Pax2 (FlyFactorSurvey ID Sv_SOLEXA_5), respectively. The 8-mer occurrence score is defined by the most frequently occurring 8-mer within each B1H hit. Numbers above the bars indicate the number of unique 8-mers within each bar. **(C, D)** Scatter plots demonstrating positive relationship between 8-mer occurrence score (log-transformed) of each B1H hit and the second-highest 8-mer PBM E-score per B1H hit. E-scores for Pax2 and Sens were derived from *D*. *rerio* Pax2b PBM (CISBP Accession M1499_1.02) and *M*. *musculus* Gfi1 PBM (Uniprobe Accession UP00591), respectively. R-value shown is the Spearman’s rank correlation and p-value of correlation is noted. The blue line (slope = 1, y-intercept = 0) is shown as a reference of a perfect correlation. **(E, F)** Boxplots demonstrate the distribution of second-highest E-scores per B1H hit in each 8-mer occurrence score quartiles. Note that as quartile of 8-mer occurrence score decreases, the distribution of E-score also decreases. Kruskal-Wallis test was used to do one-way variance test (indicated on plots). Each quartile was compared to the entire pool of B1H hits using t-test and p-values were Bonferroni adjusted for multiple comparisons.
(TIF)

S12 Fig Comparison of information content of Pax2 and Sens PWMs generated by sub-sampling quartiles of B1H derived sequences. Information content of Pax2 (A) and Sens (B) PWMs generated from FlyFactorSurvey B1H data [24]. For **Fig 7**, each B1H sequence was assigned an affinity score derived from the occurrence of 8-mers relative to the whole pool of B1H sequences (see **Methods** for details). The B1H sequences were grouped into 4 quartiles based on this affinity score and 100 PWMs were generated by iteratively sampling 50 B1H sequences from each quartile. At top, we show a representative PWM logo from each Quartile. Note that as the 8-mer occurrence score increases, the information content of the derived PWMs also increases (bottom).
(TIF)

S13 Fig Performance of B1H-derived Sens PWMs on Gfi1/Gfi1b ChIP datasets. B1H sequences [24] for Sens were binned into quartiles based on predicted affinity and 100 PWMs were generated from each quartile along with 100 additional PWMs generated by sampling the entire B1H dataset (the same Sens PWMs shown in **Figs 7 and S12**). The ability of these PWMs to discriminate Gfi1 and Gfi1b ChIP-seq peaks from an equal number of random genomic regions was assessed using the AUROC metric. The NCBI GEO accession number for each ChIP dataset is given above each plot: GSM1229967 [32], GSM1278242 [34], GSM1448829 [35], GSM1692853 [36], GSM1692854 [36], GSM1708653 [37], GSM1721242 [38], GSM2231903 [39], GSM2231904 [39], GSM2423488 [40]. All violin plots are scaled to have the same width. Statistical analysis was performed using Kurskal-Wallis test followed by a post-hoc pairwise Mann-Whitney U test. P-values were Bonferroni-adjusted due to multiple comparisons arising from groups of PWMs (n.s. p ≥ 0.05; * p < 0.05; ** p < 0.01, *** p < 0.001).
(TIF)

S14 Fig Conservation of low PWM scores for the Pax2 and Sens binding sites in *RhoA* across Drosophilids. **(A)** Alignment of *RhoA* sequence among 22 *Drosophilid* species (derived from UCSC Multiz track alignment) [78]. **(B)** The low Sens PWM score for the *RhoA* Pax2 binding site is conserved. **(C)** The low Pax2 PWM score for the *RhoA* Sens binding site is conserved.
(TIF)

S1 Data Original position-weight and position-frequency matrices downloaded from FlyFactorSurvey and Nitta et al, 2015. (TXT)

S2 Data FastQC analysis of sequencing reads for Gfi1 ChIP-seq in mouse innate type-2 lymophcytes (ILC-2) (SRP029908, Spooner et al, 2013). (HTML)

S3 Data FastQC analysis of sequencing reads for Gfi1b ChIP-seq in mouse haematopoietic progenitor cell line (HPC-7) (SRP002575, Wilson et al, 2010). (HTML)

S4 Data FastQC analysis of sequencing reads for Gfi1 ChIP-seq in mouse granulocyte-monocyte progenitors (SRP059846, Olsson et al, 2016). (HTML)

S5 Data FastQC analysis of sequencing reads for Gfi1b ChIP-seq in mouse erythroleukemia cells (SRP045060, Stadhouders et al, 2015). (HTML)

S6 Data Raw measurements used for creating plots in Figs 2 to 5. (XLSX)
