# Supplementary figures and images for: Degenerate Pax2 and Senseless binding motifs improve detection of low-affinity sites required for enhancer specificity
Source: PLoS Genet. 2018 Apr 4;14(4):e1007289. doi: 10.1371/journal.pgen.1007289 (PMC5902045; doi:10.1371/journal.pgen.1007289)

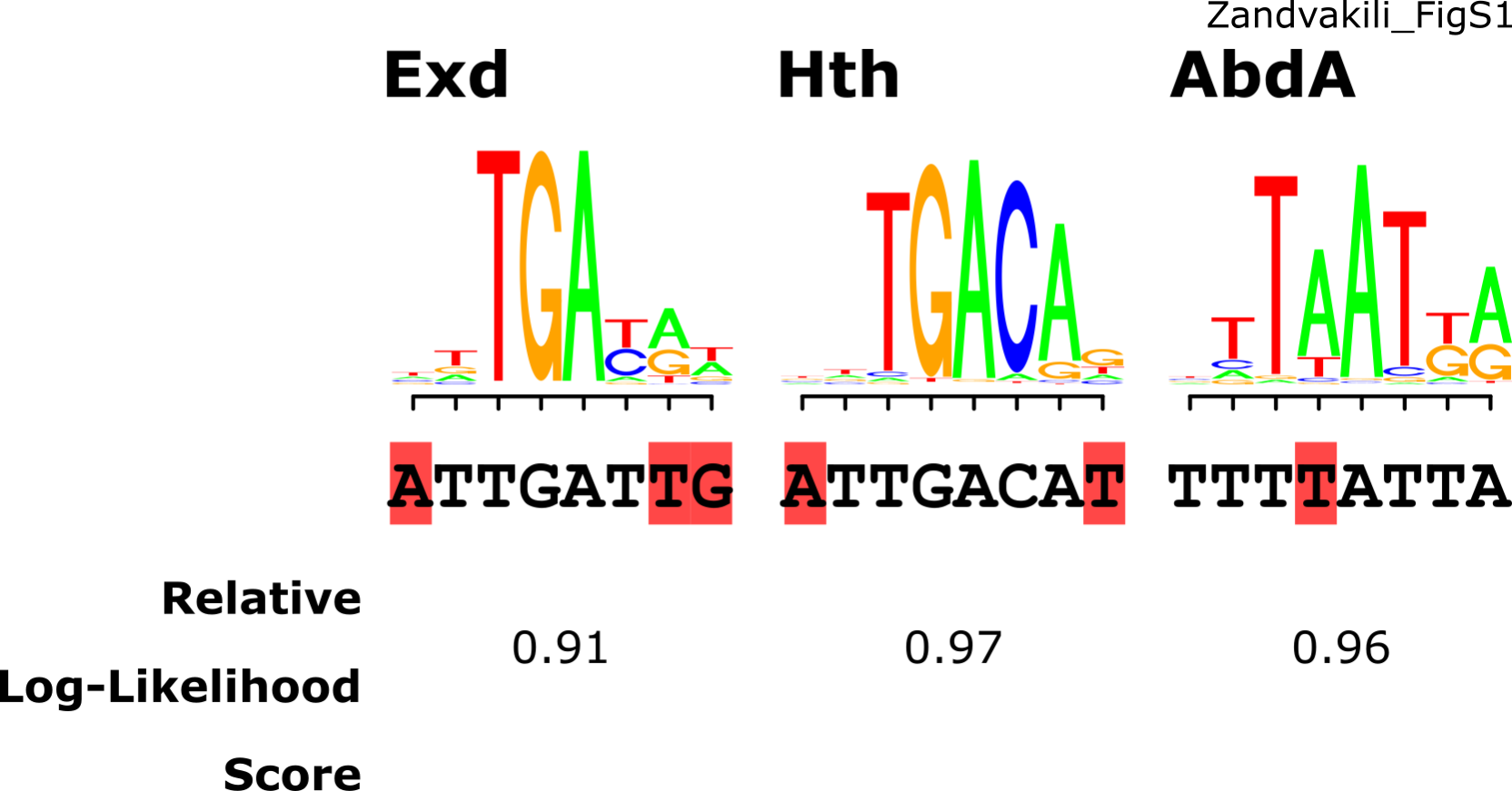

Supplement: S1 Fig — PWMs were downloaded from the FlyFactorSurvey website and aligned to the RhoA sequence. The RLL score for each RhoA transcription factor site is listed. (TIF) [file pgen.1007289.s001.tif]

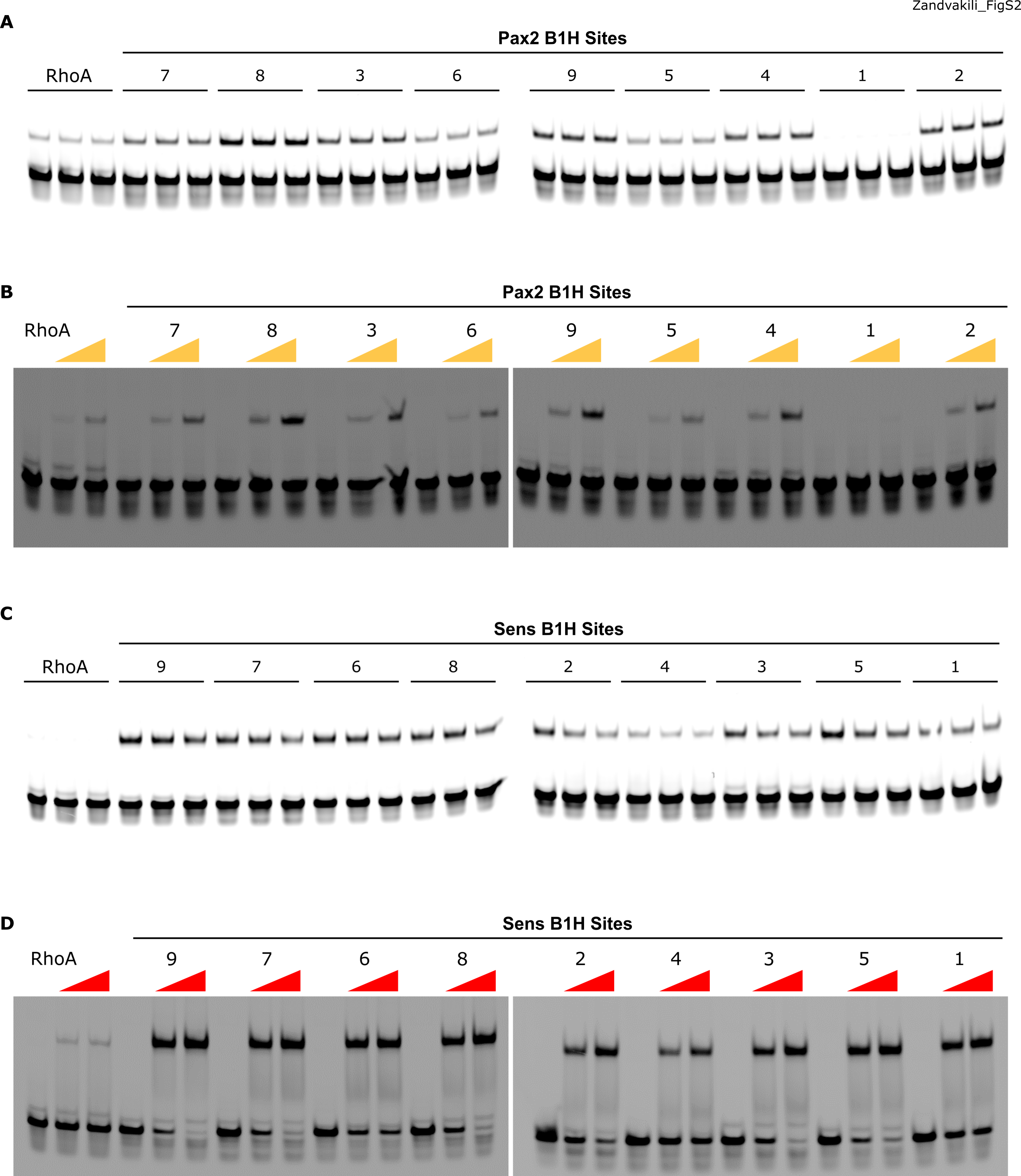

Supplement: S2 Fig — EMSA analysis for the binding of Pax2 (A-B) and Sens (C-D) to wildtype RhoA probes or RhoA probes in which the Sens or Pax2 binding sites have been replaced with B1H hits. Sequence IDs correspond to sequences in Fig 2A and 2D. (A, C) EMSAs performed, respectively, with 48 ng of Pax2 or 106 ng of Sens in triplicate. These EMSAs were quantified to produce graphs in Fig 2C and 2F. (B, D) Uncropped versions of gels shown in Fig 2B and 2E. (TIF) [file pgen.1007289.s002.tif]

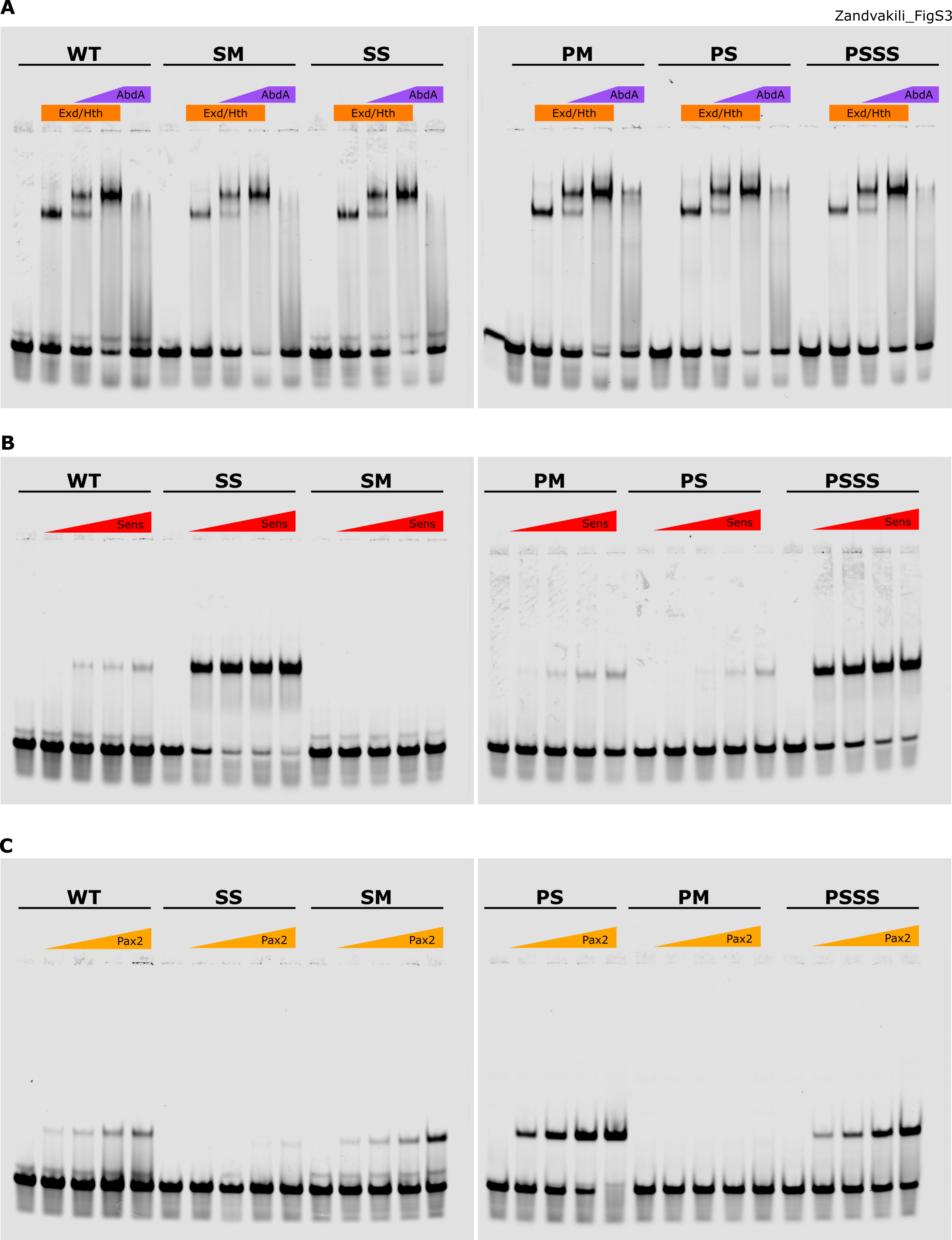

Supplement: S3 Fig — EMSA analysis for the binding of Exd/Hth/Hox (A), Sens (B), or Pax2 (C) to probes carrying each RhoA variant shown in Figs 3and 4. The protein concentrations used in were as follows: Exd/Hth: 59.2 ng; AbdA: 94.5 and 189 ng; Sens: 0, 23.5, 57, 114, and 228 ng; Pax2: 0, 10.25, 20.5, 41, and 82 ng. These panels show the complete gels, whereas relevant portions of these gels are shown in Figs 3and 4. (TIF) [file pgen.1007289.s003.tif]

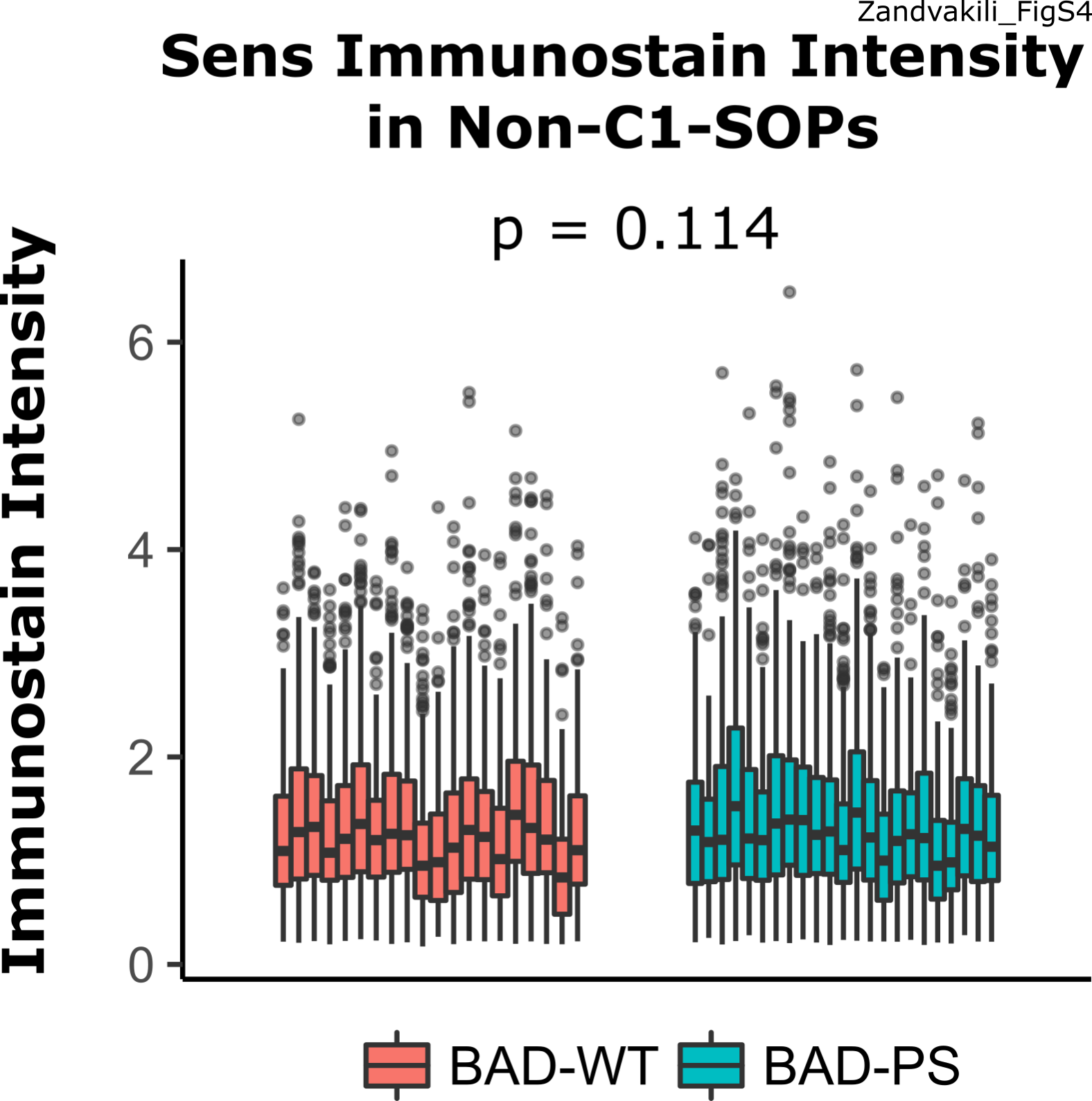

Supplement: S4 Fig — Each boxplot represents Sens levels in non-C1 Sens+ nuclei per embryo. Note, no significant difference in Sens levels were observed between reporter genotypes. Statistical analysis was conducted using the Welch’s T-test to compare mean reporter activity per embryo between the two genotypes. (TIF) [file pgen.1007289.s004.tif]

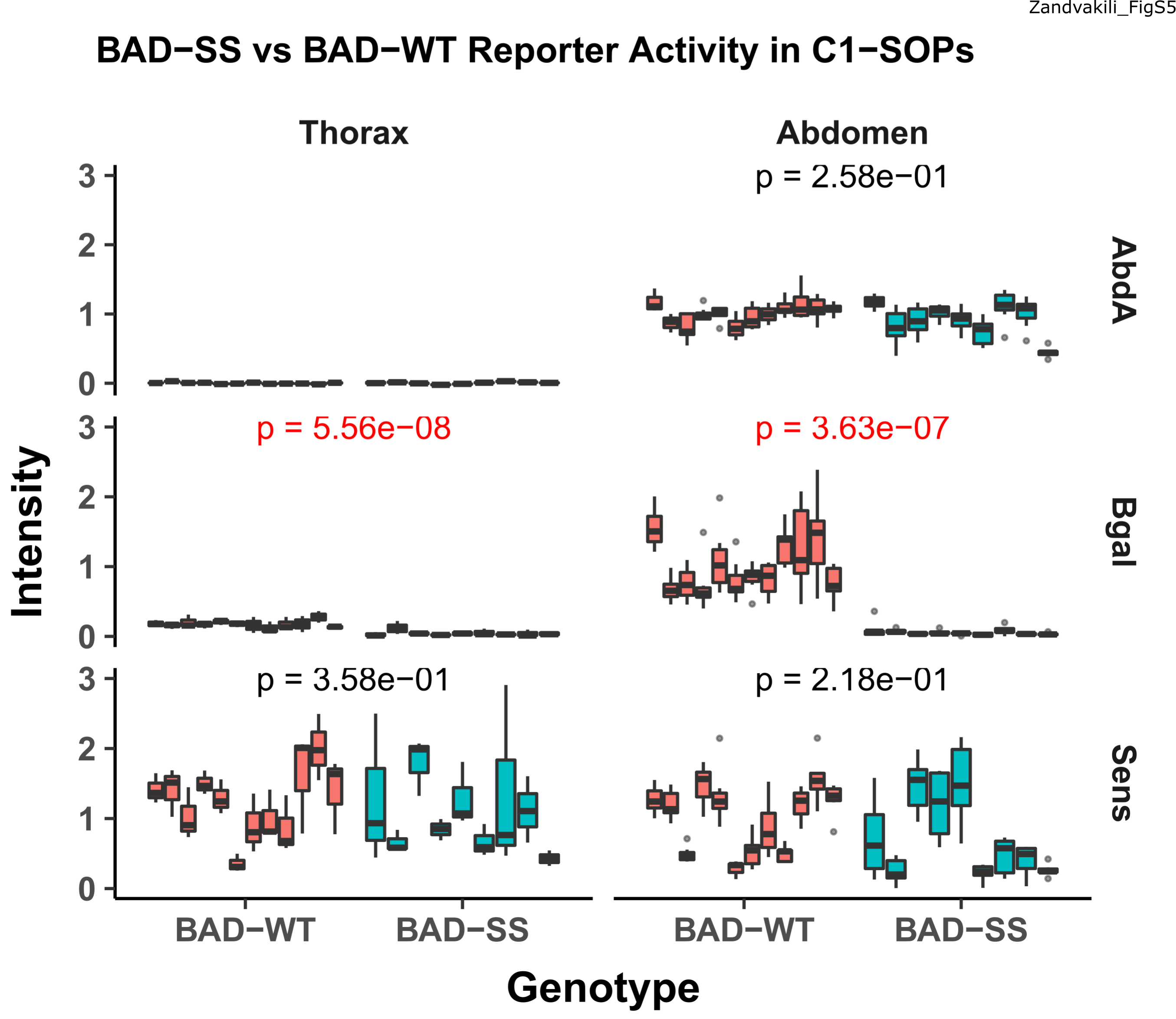

Supplement: S5 Fig — Each boxplot represents the indicated AbdA, Sens, and β-gal levels in either thoracic or abdominal C1-SOPs. Note, no significant difference in Sens or AbdA levels were observed between reporter genotypes. Statistical analysis was conducted using the Welch’s T-test to compare mean reporter activity per embryo between the two genotypes. (TIF) [file pgen.1007289.s005.tif]

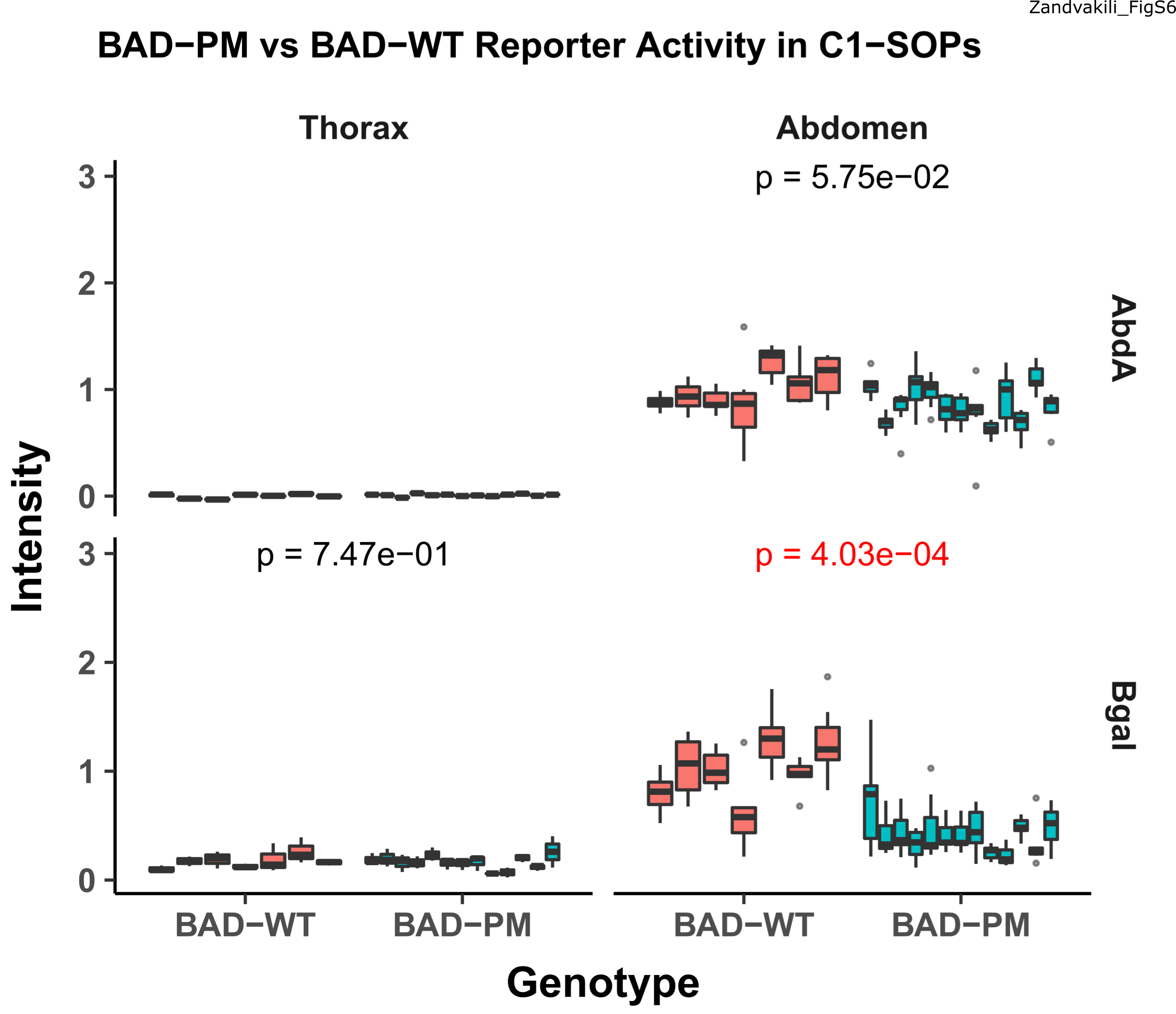

Supplement: S6 Fig — Each boxplot represents the indicated AbdA and β-gal levels in either thoracic or abdominal C1-SOPs. Note, there is a significant difference in abdominal β-gal levels, but no significant difference in AbdA levels between reporter genotypes. Statistical analysis was conducted using the Welch’s T-test to compare mean reporter activity per embryo between the two genotypes. (TIF) [file pgen.1007289.s006.tif]

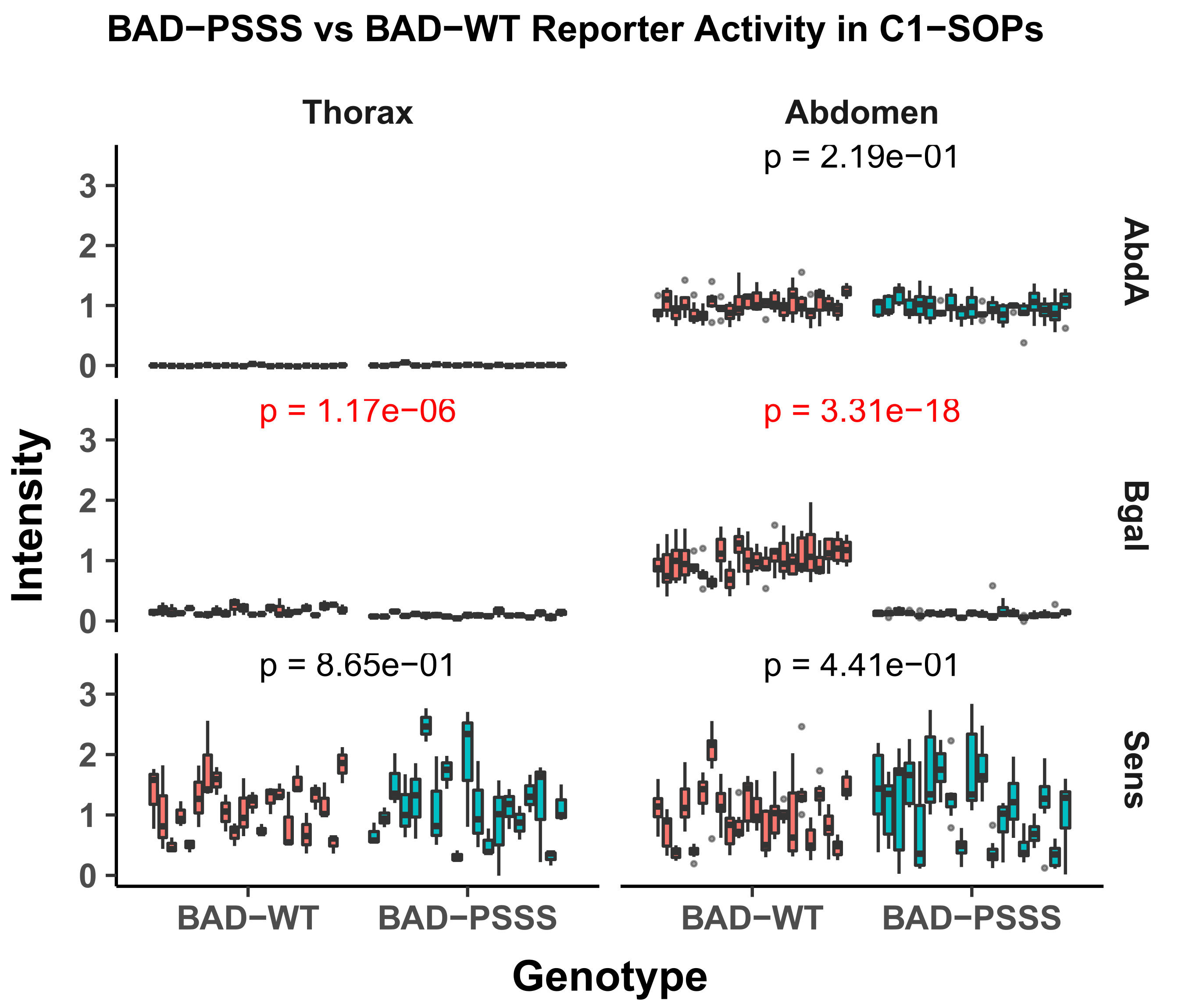

Supplement: S7 Fig — Each boxplot represents the indicated AbdA, Sens and β-gal levels in either thoracic or abdominal C1-SOPs. Note, there is a significant difference in abdominal β-gal levels, but no significant difference in AbdA or Sens levels were observed between reporter genotypes. Statistical analysis was conducted using the Welch’s T-test to compare mean reporter activity per embryo between the two genotypes. (TIF) [file pgen.1007289.s007.tif]

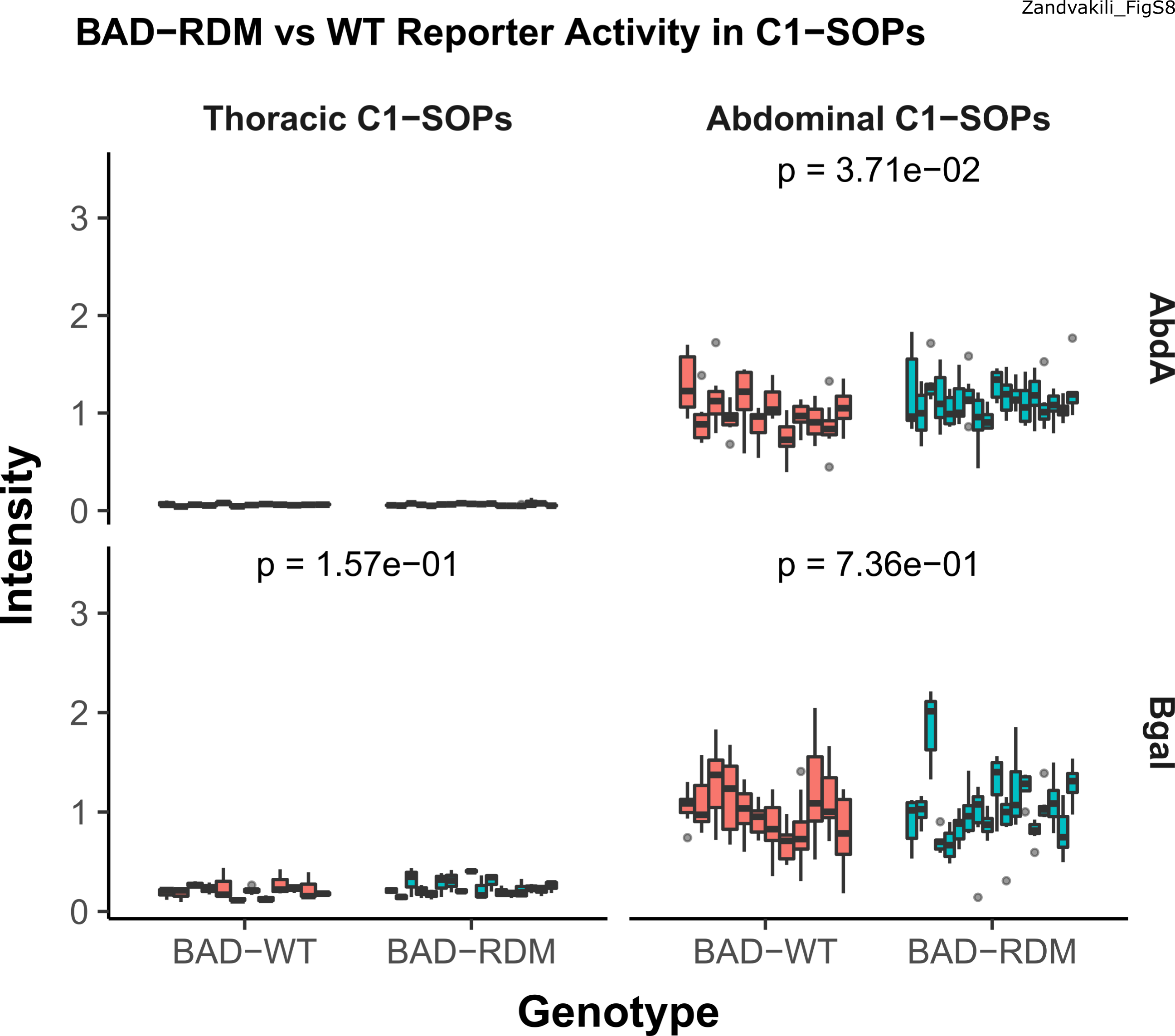

Supplement: S8 Fig — Each boxplot represents the indicated AbdA and β-gal levels in either thoracic or abdominal C1-SOPs. Note, no significant difference in levels were observed between reporter genotypes. Statistical analysis was conducted using the Welch’s T-test to compare mean reporter activity per embryo between the two genotypes. (TIF) [file pgen.1007289.s008.tif]

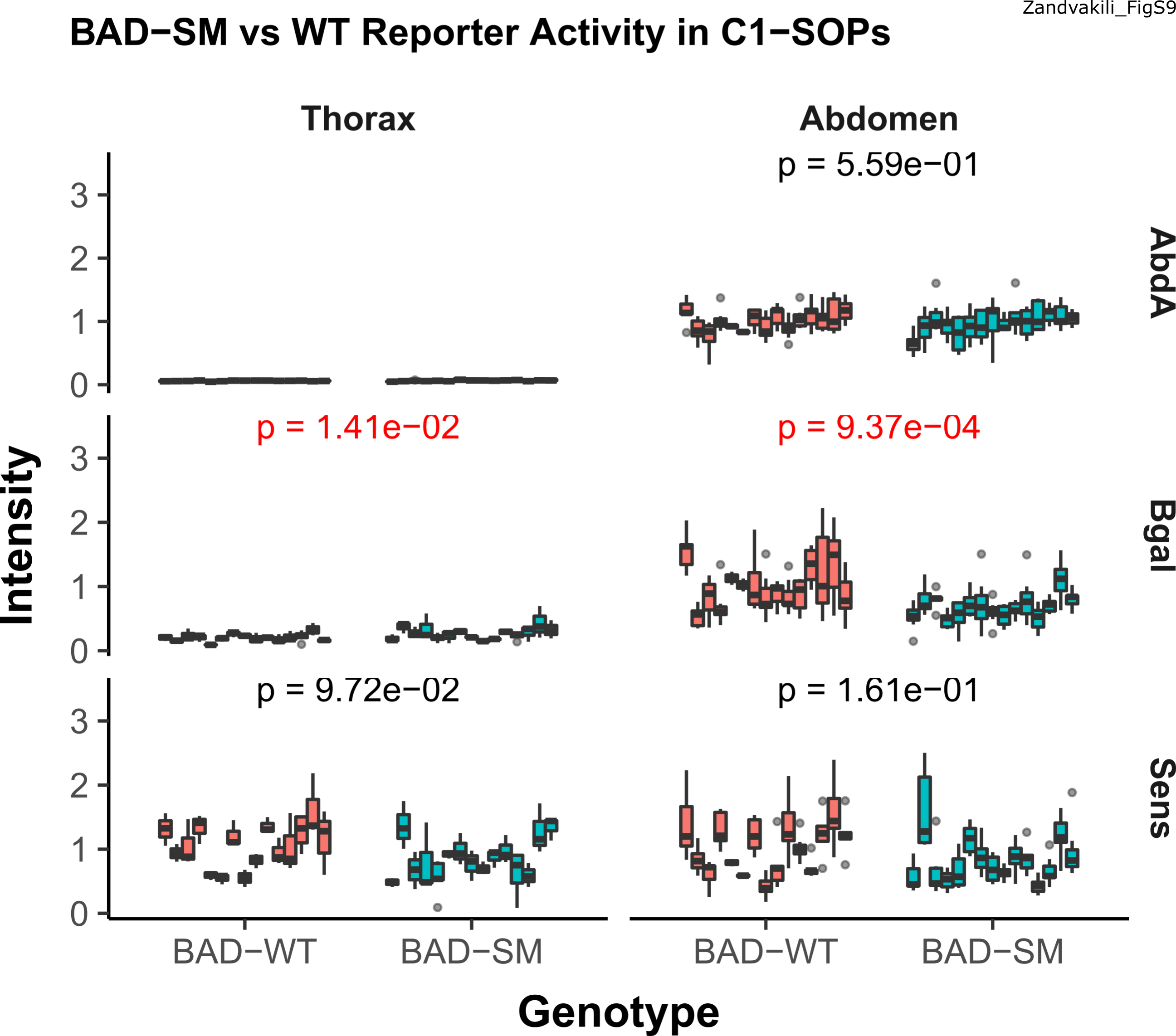

Supplement: S9 Fig — Each boxplot represents the indicated AbdA, Sens and β-gal levels in either thoracic or abdominal C1-SOPs. Note there is a significant difference in β-gal levels, but no significant difference in AbdA or Sens levels were observed between reporter genotypes. Statistical analysis was conducted using the Welch’s T-test to compare mean reporter activity per embryo between the two genotypes. (TIF) [file pgen.1007289.s009.tif]

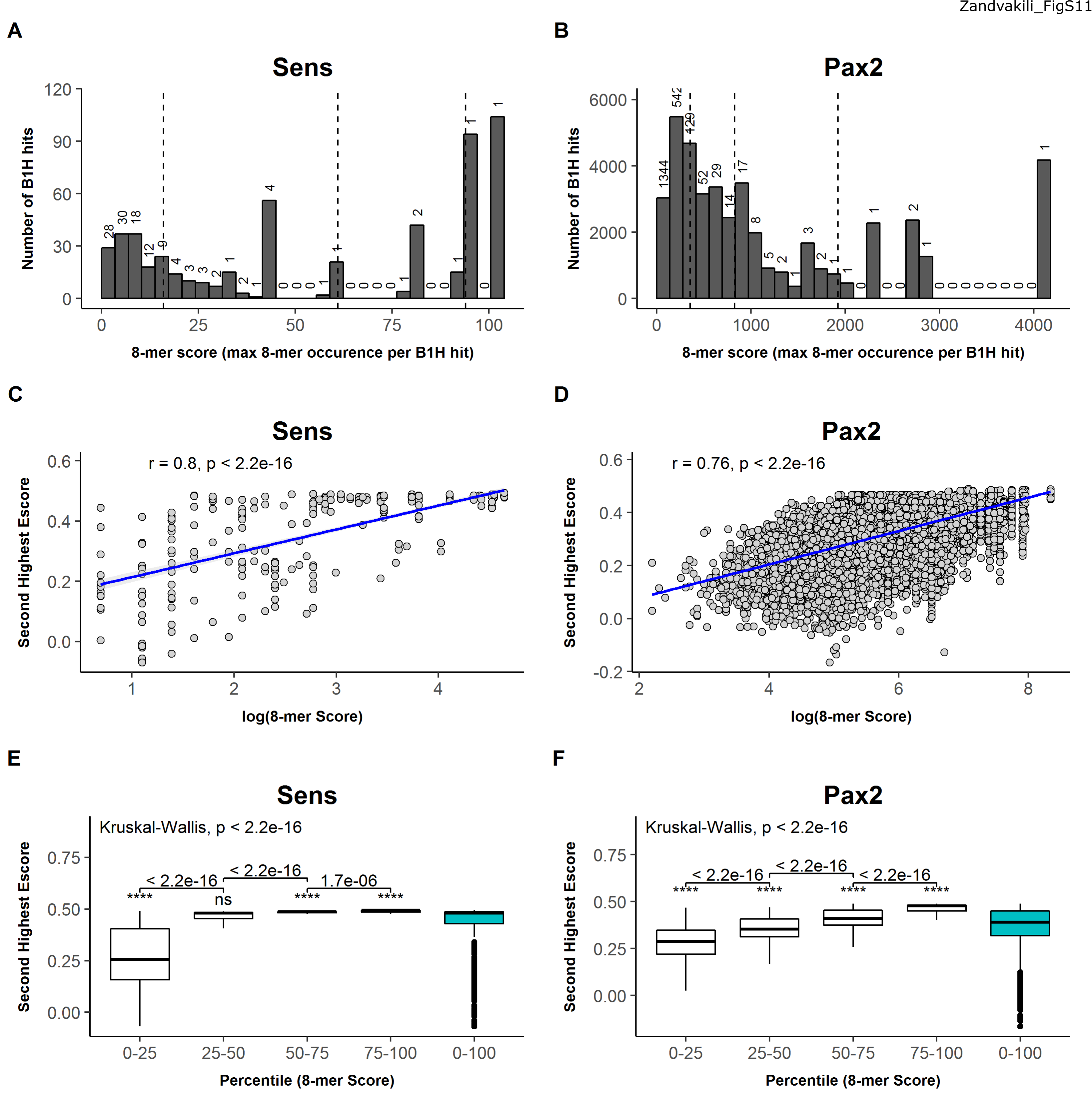

Supplement: S11 Fig — (A, B) Histograms displaying the distribution of 8-mer occurrence scores of B1H hits for Sens (FlyFactorSurvey ID Sens_SOLEXA_5) and Pax2 (FlyFactorSurvey ID Sv_SOLEXA_5), respectively. The 8-mer occurrence score is defined by the most frequently occurring 8-mer within each B1H hit. Numbers above the bars indicate the number of unique 8-mers within each bar. (C, D) Scatter plots demonstrating positive relationship between 8-mer occurrence score (log-transformed) of each B1H hit and the second-highest 8-mer PBM E-score per B1H hit. E-scores for Pax2 and Sens were derived from D. rerio Pax2b PBM (CISBP Accession M1499_1.02) and M. musculus Gfi1 PBM (Uniprobe Accession UP00591), respectively. R-value shown is the Spearman’s rank correlation and p-value of correlation is noted. The blue line (slope = 1, y-intercept = 0) is shown as a reference of a perfect correlation. (E, F) Boxplots demonstrate the distribution of second-highest E-scores per B1H hit in each 8-mer occurrence score quartiles. Note that as quartile of 8-mer occurrence score decreases, the distribution of E-score also decreases. Kruskal-Wallis test was used to do one-way variance test (indicated on plots). Each quartile was compared to the entire pool of B1H hits using t-test and p-values were Bonferroni adjusted for multiple comparisons. (TIF) [file pgen.1007289.s011.tif]

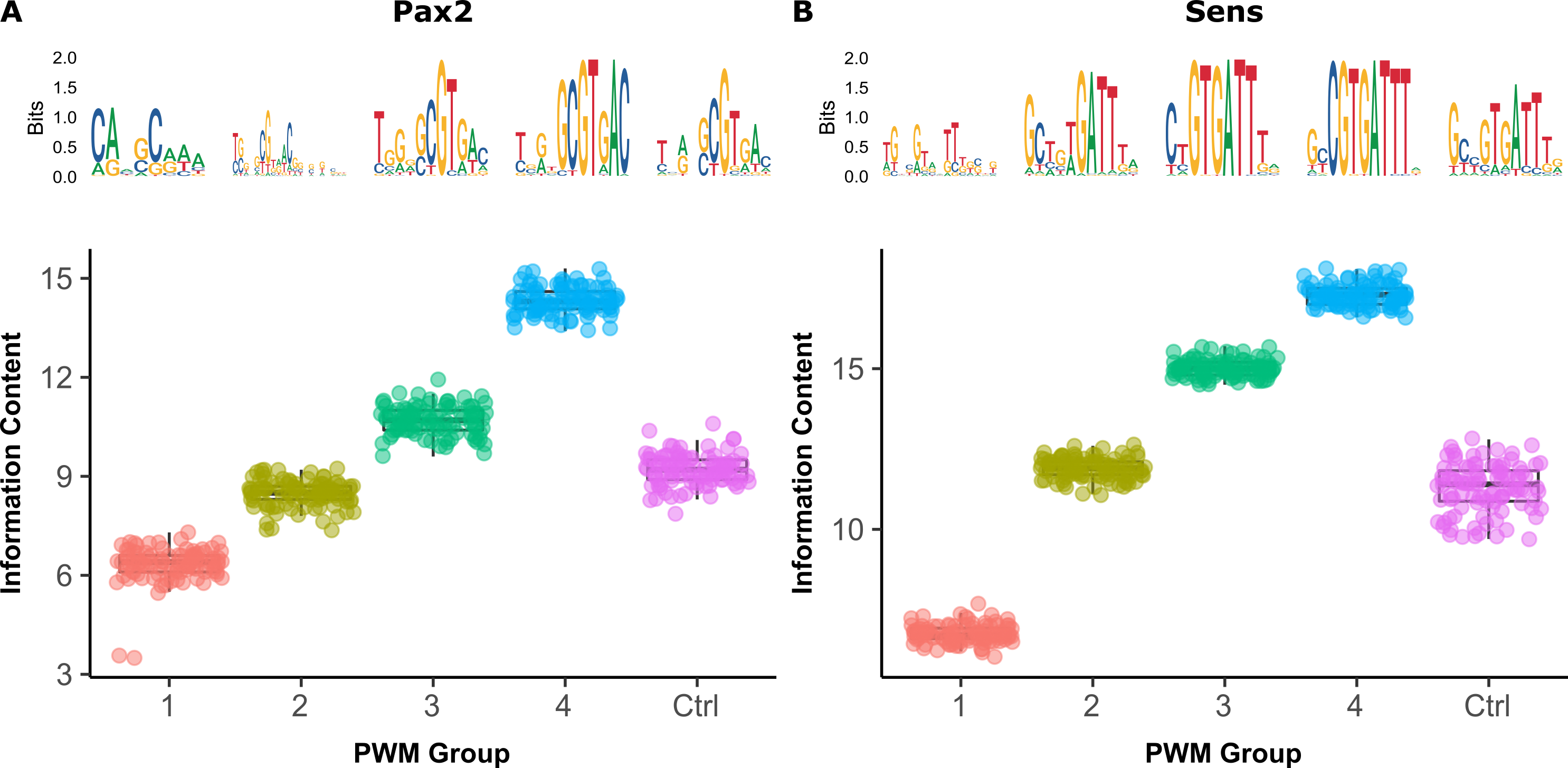

Supplement: S12 Fig — Information content of Pax2 (A) and Sens (B) PWMs generated from FlyFactorSurvey B1H data [24]. For Fig 7, each B1H sequence was assigned an affinity score derived from the occurrence of 8-mers relative to the whole pool of B1H sequences (see Methods for details). The B1H sequences were grouped into 4 quartiles based on this affinity score and 100 PWMs were generated by iteratively sampling 50 B1H sequences from each quartile. At top, we show a representative PWM logo from each Quartile. Note that as the 8-mer occurrence score increases, the information content of the derived PWMs also increases (bottom). (TIF) [file pgen.1007289.s012.tif]

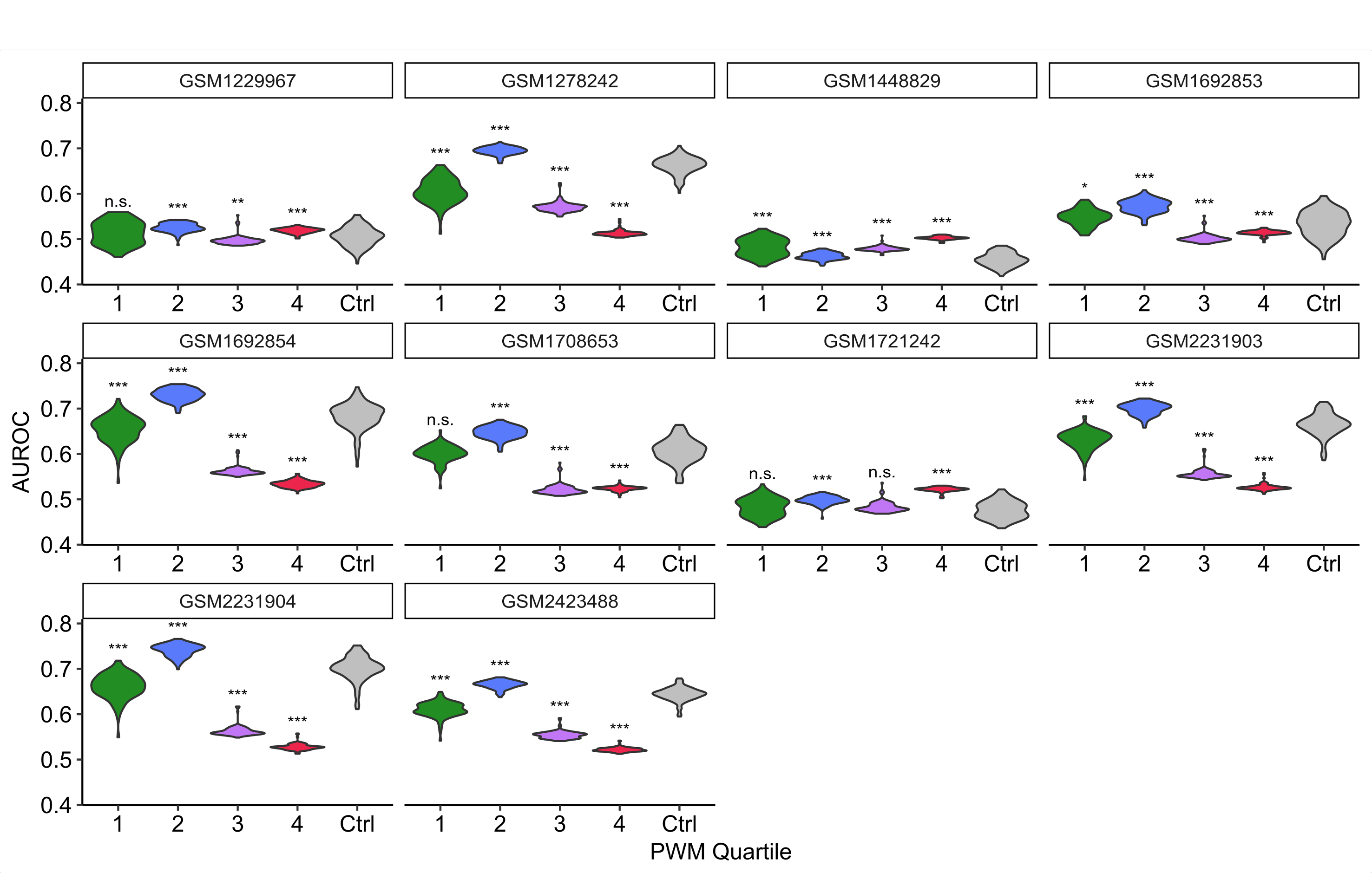

Supplement: S13 Fig — B1H sequences [24] for Sens were binned into quartiles based on predicted affinity and 100 PWMs were generated from each quartile along with 100 additional PWMs generated by sampling the entire B1H dataset (the same Sens PWMs shown in Figs 7 and S12). The ability of these PWMs to discriminate Gfi1 and Gfi1b ChIP-seq peaks from an equal number of random genomic regions was assessed using the AUROC metric. The NCBI GEO accession number for each ChIP dataset is given above each plot: GSM1229967 [32], GSM1278242 [34], GSM1448829 [35], GSM1692853 [36], GSM1692854 [36], GSM1708653 [37], GSM1721242 [38], GSM2231903 [39], GSM2231904 [39], GSM2423488 [40]. All violin plots are scaled to have the same width. Statistical analysis was performed using Kurskal-Wallis test followed by a post-hoc pairwise Mann-Whitney U test. P-values were Bonferroni-adjusted due to multiple comparisons arising from groups of PWMs (n.s. p ≥ 0.05; * p < 0.05; ** p < 0.01, *** p < 0.001). (TIF) [file pgen.1007289.s013.tif]

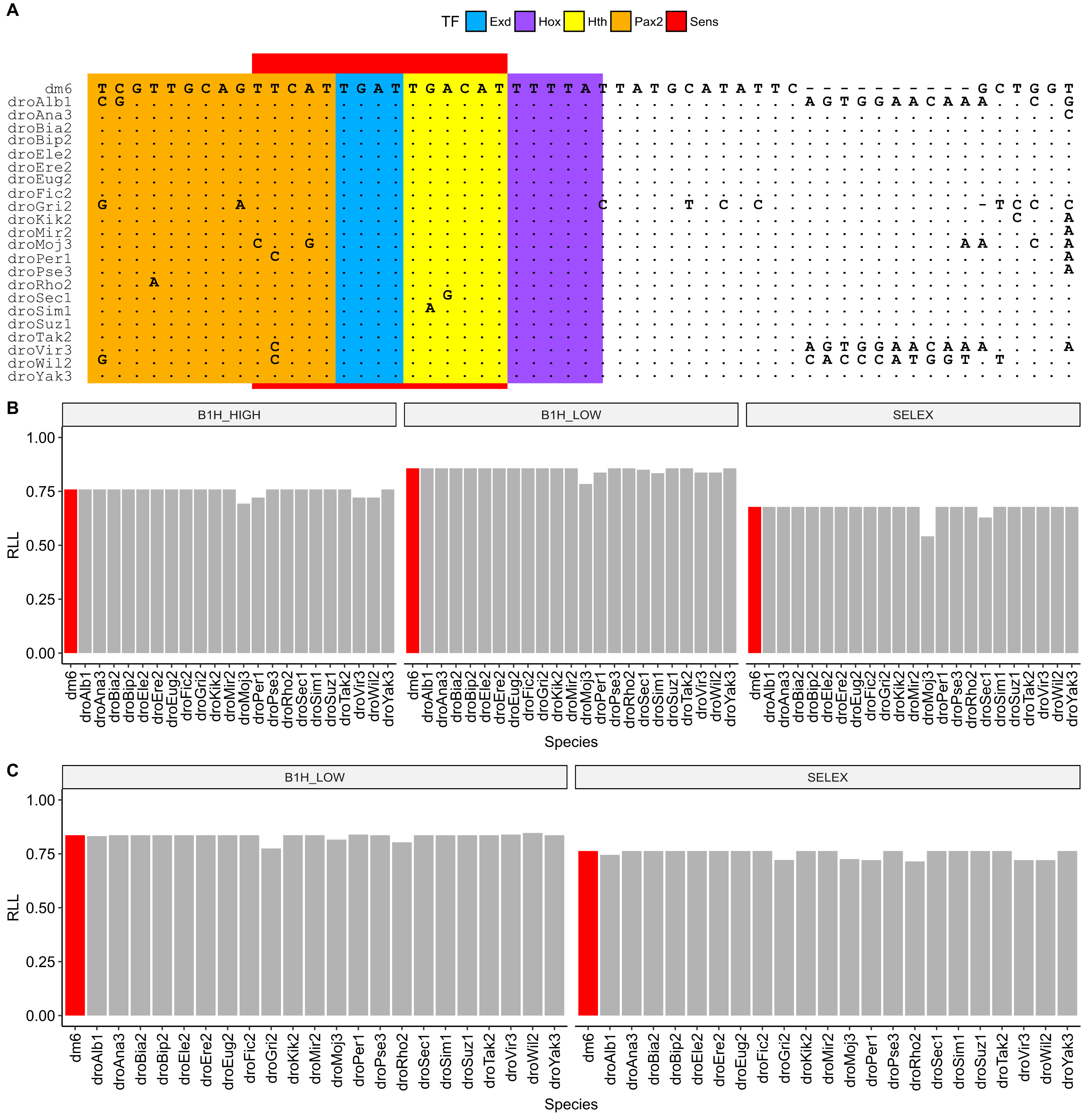

Supplement: S14 Fig — (A) Alignment of RhoA sequence among 22 Drosophilid species (derived from UCSC Multiz track alignment) [78]. (B) The low Sens PWM score for the RhoA Pax2 binding site is conserved. (C) The low Pax2 PWM score for the RhoA Sens binding site is conserved. (TIF) [file pgen.1007289.s014.tif]
