# Supplementary material for: Degenerate Pax2 and Senseless binding motifs improve detection of low-affinity sites required for enhancer specificity
Source: PLoS Genet. 2018 Apr 4;14(4):e1007289. doi: 10.1371/journal.pgen.1007289 (PMC5902045; doi:10.1371/journal.pgen.1007289)
Supplement: S2 Data — (HTML) [file pgen.1007289.s016.html]

Extract\_reads FastQC Report 

FastQC Report

Tue 21 Mar 2017  
Extract\_reads

## Summary

- Basic Statistics
- Per base sequence quality
- Per tile sequence quality
- Per sequence quality scores
- Per base sequence content
- Per sequence GC content
- Per base N content
- Sequence Length Distribution
- Sequence Duplication Levels
- Overrepresented sequences
- Adapter Content
- Kmer Content

## Basic Statistics

| Measure | Value |
| --- | --- |
| Filename | Extract\_reads |
| File type | Conventional base calls |
| Encoding | Sanger / Illumina 1.9 |
| Total Sequences | 20723593 |
| Sequences flagged as poor quality | 0 |
| Sequence length | 50 |
| %GC | 48 |

## Per base sequence quality

## Per tile sequence quality

## Per sequence quality scores

## Per base sequence content

## Per sequence GC content

## Per base N content

## Sequence Length Distribution

## Sequence Duplication Levels

## Overrepresented sequences

| Sequence | Count | Percentage | Possible Source |
| --- | --- | --- | --- |
| GATCGGAAGAGCGGTTCAGCAGGAATGCCGAGACCGGGAGCTAATCTCGT | 24225 | 0.11689575258498851 | Illumina Paired End PCR Primer 2 (97% over 36bp) |

## Adapter Content

## Kmer Content

| Sequence | Count | PValue | Obs/Exp Max | Max Obs/Exp Position |
| --- | --- | --- | --- | --- |
| AATCTCG | 4330 | 0.0 | 30.874418 | 43 |
| ATCTCGT | 4975 | 0.0 | 26.960064 | 44 |
| GCTAATC | 5100 | 0.0 | 26.602036 | 40 |
| ACCGGGA | 5210 | 0.0 | 26.034369 | 33 |
| GATCGGA | 6560 | 0.0 | 24.83014 | 1 |
| AGACCGG | 5490 | 0.0 | 24.828049 | 31 |
| CCGGGAG | 5495 | 0.0 | 24.684029 | 34 |
| ATCGGAA | 6700 | 0.0 | 24.582247 | 2 |
| CGGGAGC | 5520 | 0.0 | 24.572353 | 35 |
| CCGAGAC | 5615 | 0.0 | 24.084394 | 28 |
| GAGACCG | 5720 | 0.0 | 23.758244 | 30 |
| TCGGAAG | 7140 | 0.0 | 23.344553 | 3 |
| GAGCGGT | 7285 | 0.0 | 22.877697 | 9 |
| GACCGGG | 5945 | 0.0 | 22.85364 | 32 |
| AAGAGCG | 7660 | 0.0 | 21.729 | 7 |
| AGCGGTT | 7750 | 0.0 | 21.703629 | 10 |
| CTAATCT | 6350 | 0.0 | 21.572767 | 41 |
| CGGTTCA | 7975 | 0.0 | 21.118515 | 12 |
| AGAGCGG | 7920 | 0.0 | 21.015675 | 8 |
| CGAGACC | 6480 | 0.0 | 20.937447 | 29 |

Produced by FastQC (version 0.11.5)
