# Supplementary material for: Degenerate Pax2 and Senseless binding motifs improve detection of low-affinity sites required for enhancer specificity
Source: PLoS Genet. 2018 Apr 4;14(4):e1007289. doi: 10.1371/journal.pgen.1007289 (PMC5902045; doi:10.1371/journal.pgen.1007289)
Supplement: S3 Data — (HTML) [file pgen.1007289.s017.html]

Extract\_reads FastQC Report 

FastQC Report

Tue 21 Mar 2017  
Extract\_reads

## Summary

- Basic Statistics
- Per base sequence quality
- Per tile sequence quality
- Per sequence quality scores
- Per base sequence content
- Per sequence GC content
- Per base N content
- Sequence Length Distribution
- Sequence Duplication Levels
- Overrepresented sequences
- Adapter Content
- Kmer Content

## Basic Statistics

| Measure | Value |
| --- | --- |
| Filename | Extract\_reads |
| File type | Conventional base calls |
| Encoding | Sanger / Illumina 1.9 |
| Total Sequences | 16831158 |
| Sequences flagged as poor quality | 0 |
| Sequence length | 37 |
| %GC | 48 |

## Per base sequence quality

## Per tile sequence quality

## Per sequence quality scores

## Per base sequence content

## Per sequence GC content

## Per base N content

## Sequence Length Distribution

## Sequence Duplication Levels

## Overrepresented sequences

| Sequence | Count | Percentage | Possible Source |
| --- | --- | --- | --- |
| GATCGGAAGAGCTCGTATGCCGTCTTCTGCTTGAAAA | 102090 | 0.6065536310692349 | Illumina Single End Adapter 1 (100% over 33bp) |
| GATCGGAAGAGCGGTTCAGCAGGAATGCCGAGATCGG | 21471 | 0.1275669802398623 | Illumina Paired End PCR Primer 2 (97% over 36bp) |

## Adapter Content

## Kmer Content

| Sequence | Count | PValue | Obs/Exp Max | Max Obs/Exp Position |
| --- | --- | --- | --- | --- |
| TCGGAAG | 47775 | 0.0 | 27.974857 | 3 |
| CGGAAGA | 44510 | 0.0 | 27.86347 | 4 |
| ATCGGAA | 48315 | 0.0 | 27.746346 | 2 |
| GATCGGA | 48585 | 0.0 | 27.606752 | 1 |
| GACTCGT | 4165 | 0.0 | 27.428328 | 9 |
| ACTCGTA | 4205 | 0.0 | 27.055767 | 10 |
| GAGTCGT | 3665 | 0.0 | 26.772259 | 9 |
| AGTCGTA | 3740 | 0.0 | 26.277225 | 10 |
| AGAGTCG | 3855 | 0.0 | 25.817205 | 8 |
| GAGCTCG | 24755 | 0.0 | 25.627855 | 9 |
| AGACTCG | 4530 | 0.0 | 25.586496 | 8 |
| GTCGTAT | 3855 | 0.0 | 25.371555 | 11 |
| GAAGAGC | 34255 | 0.0 | 24.141191 | 6 |
| AAGAGCT | 30525 | 0.0 | 23.805357 | 7 |
| GGAAGAG | 42730 | 0.0 | 23.248274 | 5 |
| AGAGCTC | 28630 | 0.0 | 23.076103 | 8 |
| AGCTCGT | 27645 | 0.0 | 22.920424 | 10 |
| CGGTTCA | 3295 | 0.0 | 22.179026 | 12 |
| GAGCGGT | 3420 | 0.0 | 21.734966 | 9 |
| GCTCGTA | 29600 | 0.0 | 21.438313 | 11 |

Produced by FastQC (version 0.11.5)
