# Supplementary material for: Degenerate Pax2 and Senseless binding motifs improve detection of low-affinity sites required for enhancer specificity
Source: PLoS Genet. 2018 Apr 4;14(4):e1007289. doi: 10.1371/journal.pgen.1007289 (PMC5902045; doi:10.1371/journal.pgen.1007289)
Supplement: S4 Data — (HTML) [file pgen.1007289.s018.html]

Extract\_reads FastQC Report 

FastQC Report

Tue 21 Mar 2017  
Extract\_reads

## Summary

- Basic Statistics
- Per base sequence quality
- Per tile sequence quality
- Per sequence quality scores
- Per base sequence content
- Per sequence GC content
- Per base N content
- Sequence Length Distribution
- Sequence Duplication Levels
- Overrepresented sequences
- Adapter Content
- Kmer Content

## Basic Statistics

| Measure | Value |
| --- | --- |
| Filename | Extract\_reads |
| File type | Conventional base calls |
| Encoding | Sanger / Illumina 1.9 |
| Total Sequences | 49361770 |
| Sequences flagged as poor quality | 0 |
| Sequence length | 50 |
| %GC | 44 |

## Per base sequence quality

## Per tile sequence quality

## Per sequence quality scores

## Per base sequence content

## Per sequence GC content

## Per base N content

## Sequence Length Distribution

## Sequence Duplication Levels

## Overrepresented sequences

No overrepresented sequences

## Adapter Content

## Kmer Content

| Sequence | Count | PValue | Obs/Exp Max | Max Obs/Exp Position |
| --- | --- | --- | --- | --- |
| TCTCGTA | 3920 | 0.0 | 26.15294 | 44 |
| ACCGCTT | 4245 | 0.0 | 24.409115 | 33 |
| ATGCCGA | 14210 | 0.0 | 23.098583 | 25 |
| AGACCGC | 4560 | 0.0 | 22.626196 | 31 |
| CCGCTTG | 4625 | 0.0 | 22.546377 | 34 |
| CGCTTGT | 4615 | 0.0 | 22.50012 | 35 |
| AATCTCG | 4670 | 0.0 | 22.376818 | 42 |
| CGAGACC | 4870 | 0.0 | 21.366919 | 29 |
| TGCCGAG | 15520 | 0.0 | 21.27647 | 26 |
| GCCGAGA | 14770 | 0.0 | 20.971615 | 27 |
| GACCGCT | 5075 | 0.0 | 20.590267 | 32 |
| ATCTCGT | 5185 | 0.0 | 19.942131 | 43 |
| GAGACCG | 5505 | 0.0 | 18.942215 | 30 |
| CCGAGAC | 5610 | 0.0 | 18.901396 | 28 |
| CGAGATC | 10865 | 0.0 | 18.60782 | 29 |
| GAGATCG | 11105 | 0.0 | 18.14624 | 30 |
| GAGCGGT | 18740 | 0.0 | 17.902365 | 9 |
| GATCGGA | 18865 | 0.0 | 17.807528 | 1 |
| CCGAGAT | 11485 | 0.0 | 17.7757 | 28 |
| GCGGTTC | 19080 | 0.0 | 17.525806 | 11 |

Produced by FastQC (version 0.11.5)
