# Supplementary material for: Degenerate Pax2 and Senseless binding motifs improve detection of low-affinity sites required for enhancer specificity
Source: PLoS Genet. 2018 Apr 4;14(4):e1007289. doi: 10.1371/journal.pgen.1007289 (PMC5902045; doi:10.1371/journal.pgen.1007289)
Supplement: S5 Data — (HTML) [file pgen.1007289.s019.html]

SRR1531391.fastqsanger FastQC Report 

FastQC Report

Tue 21 Mar 2017  
SRR1531391.fastqsanger

## Summary

- Basic Statistics
- Per base sequence quality
- Per tile sequence quality
- Per sequence quality scores
- Per base sequence content
- Per sequence GC content
- Per base N content
- Sequence Length Distribution
- Sequence Duplication Levels
- Overrepresented sequences
- Adapter Content
- Kmer Content

## Basic Statistics

| Measure | Value |
| --- | --- |
| Filename | SRR1531391.fastqsanger |
| File type | Conventional base calls |
| Encoding | Sanger / Illumina 1.9 |
| Total Sequences | 12270949 |
| Sequences flagged as poor quality | 0 |
| Sequence length | 36 |
| %GC | 53 |

## Per base sequence quality

## Per tile sequence quality

## Per sequence quality scores

## Per base sequence content

## Per sequence GC content

## Per base N content

## Sequence Length Distribution

## Sequence Duplication Levels

## Overrepresented sequences

No overrepresented sequences

## Adapter Content

## Kmer Content

| Sequence | Count | PValue | Obs/Exp Max | Max Obs/Exp Position |
| --- | --- | --- | --- | --- |
| CTCGTAT | 210 | 4.6054993E-8 | 10.710129 | 12 |
| TCGTATG | 260 | 9.545456E-6 | 8.073955 | 13 |
| GATCGGA | 395 | 3.5312842E-4 | 5.6986094 | 1 |
| ATATAGT | 375 | 0.0011255058 | 5.6028013 | 28 |
| CGTATGC | 390 | 0.0018655069 | 5.383165 | 14 |
| ATCGGAA | 500 | 2.843594E-4 | 5.1008177 | 2 |

Produced by FastQC (version 0.11.5)
